# Infiltration by CXCL10 Secreting Macrophages Is Associated With Antitumor Immunity and Response to Therapy in Ovarian Cancer Subtypes

**DOI:** 10.3389/fimmu.2021.690201

**Published:** 2021-06-18

**Authors:** Laura Ardighieri, Francesco Missale, Mattia Bugatti, Luisa Benerini Gatta, Irene Pezzali, Matilde Monti, Stefano Gottardi, Laura Zanotti, Eliana Bignotti, Antonella Ravaggi, Germana Tognon, Franco Odicino, Stefano Calza, Yoann Missolo-Koussou, Carola Hermine Ries, Julie Helft, William Vermi

**Affiliations:** ^1^ Unit of Pathology, ASST Spedali Civili di Brescia, Brescia, Italy; ^2^ Department of Molecular and Translational Medicine, University of Brescia, Brescia, Italy; ^3^ IRCCS Ospedale Policlinico San Martino, Genova, Italy; ^4^ Diatech Pharmacogenetics srl, Jesi, Italy; ^5^ 'Angelo Nocivelli” Institute of Molecular Medicine, ASST Spedali Civili of Brescia- University of Brescia, Brescia, Italy; ^6^ Division of Obstetrics and Gynecology, ASST Spedali Civili di Brescia, Brescia, Italy; ^7^ Department of Clinical and Experimental Science, University of Brescia, Brescia, Italy; ^8^ Unit of Biostatistics, Department of Molecular and Translational Medicine, University of Brescia, Brescia, Italy; ^9^ Department of Medical Epidemiology and Biostatistics, Karolinska Institutet, Stockholm, Sweden; ^10^ Big & Open Data Innovation Laboratory, University of Brescia, Brescia, Italy; ^11^ PSL University, Institut Curie Research Center, INSERM U932 & SiRIC, Center for Cancers Immunotherapy, Translational Immunotherapy Team, Paris, France; ^12^ Dr. Carola Ries Consulting, Penzberg, Germany; ^13^ Department of Pathology and Immunology, Washington University School of Medicine, St. Louis, MO, United States

**Keywords:** ovarian cancer, macrophage, signature, CXCL10, polarization

## Abstract

Ovarian carcinomas (OCs) are poorly immunogenic and immune checkpoint inhibitors (ICIs) have offered a modest benefit. In this study, high CD3^+^ T-cells and CD163^+^ tumor-associated macrophages (TAMs) densities identify a subgroup of immune infiltrated high-grade serous carcinomas (HGSCs) with better outcomes and superior response to platinum-based therapies. On the contrary, in most clear cell carcinomas (CCCs) showing poor prognosis and refractory to platinum, a high TAM density is associated with low T cell frequency. Immune infiltrated HGSC are characterized by the 30-genes signature (OC-IS^30^) covering immune activation and IFNγ polarization and predicting good prognosis (n = 312, TCGA). Immune infiltrated HGSC contain CXCL10 producing M1-type TAM (IRF1^+^pSTAT1Y701^+^) in close proximity to T-cells. A fraction of these M1-type TAM also co-expresses TREM2. M1-polarized TAM were barely detectable in T-cell poor CCC, but identifiable across various immunogenic human cancers. Single cell RNA sequencing data confirm the existence of a tumor-infiltrating CXCL10^+^IRF1^+^STAT1^+^ M1-type TAM overexpressing antigen processing and presentation gene programs. Overall, this study highlights the clinical relevance of the CXCL10^+^IRF1^+^STAT1^+^ macrophage subset as biomarker for intratumoral T-cell activation and therefore offers a new tool to select patients more likely to respond to T-cell or macrophage-targeted immunotherapies.

## Introduction

Ovarian carcinomas (OCs) (1) represent a heterogeneous group with three main subtypes (high-grade serous carcinoma [HGSC], clear cell carcinoma [CCC] and endometrioid carcinoma [EC]) distinct by microscopic findings and molecular features. High-grade serous carcinoma (HGSC) represents the most common and lethal subtype. Patients with HGSC usually present with advanced disease involving the pelvic and peritoneal cavity associated with malignant ascites; in addition, transcoelomic metastases or distant spread can be observed at the diagnosis. Standard treatment consists of primary upfront debulking surgery followed by adjuvant cytotoxic platinum-taxane based therapy (1, 2). Most of the patients initially respond to this front-line approach; however, 70% relapses within three years. Therapy resistance mechanisms include genomic instability, epigenetic deregulation, and change in tumor microenvironment, leading to the cancer outgrowth (3, 4). A fraction of patients is refractory to platinum-based regimens and relapses early during treatment, displaying a rapid fatal course (1).

Few improvements in clinical outcomes have been obtained in OCs. Encouraging results have been achieved with the introduction of inhibitors targeting poly (ADP-ribose) polymerase (PARP), particularly effective in Homologous Recombination Deficiency (HRD) positive cases (5). HRD is detected in up to half of tumors due to inactivation of HRD genes by mutations or promoter hypermethylation (6). PARP inhibitors maintenance therapy improves progression-free survival (PFS) in platinum-sensitive newly diagnosed and recurrent OCs (7, 8). Immunotherapy based on immune checkpoint inhibitors (ICIs), has shown clinical efficacy in solid cancer (9). On the contrary, until now, the global response rate of HGSC to ICIs resulted modest, ranging from 10 to 25%, thus suggesting an urgent need for predictive biomarkers. It should be noted that OCs are characterized by low mutational burden and this could represent one of the possible explanations of lower response rate to ICIs in comparison to other cancer types (10). However, the recent combination of ICIs and PARP inhibitors in HRD^+^ OCs has shown promising results (11), suggesting higher intrinsic immunogenicity associated with the HRD group.

The composition, density, and functional orientation of the immune contexture predict patient survival and response to various treatments in different cancers (12) including OCs, the latter being traditionally considered scarcely immunogenic. A set of studies challenged this view and demonstrated that a subgroup of OCs displays a higher CD3^+^ TILs density associated with longer progression-free intervals and better survival in advanced-stage OCs (13). These observations were subsequently confirmed by others (14) and by a recent meta-analysis (15). In contrast to TILs, the clinical significance of tumor-associated macrophages (TAMs) is largely ignored with conflicting observations.

In the present study, we explored the tumor ecosystem of OCs on archival whole tumor sections. Data indicate that high density of CD3^+^ T-cells and CD163^+^ TAMs marks a consistent group of immune infiltrated HGSC, stratifies patients with different outcomes and correlates with a thirty-gene signature (OC-IS^30^) containing among others IFNγ-regulated genes. On the contrary, in most CCCs a high TAM density is not combined with a significant T-cell infiltration. By extending the analysis to The Cancer Genome Atlas, OC-IS^30^ strongly predicts a favorable outcome in a large cohort of HGSC. By immunohistochemistry for pSTAT1 and IRF1 together with RNAscope-mediated detection of CXCL10, we could identify an M1-type macrophage (Mϕ) population associated with immune infiltrated HGSC, but not CCC. We extended and confirmed these findings to other cancer types by immunohistochemistry and by an unbiased analysis of scRNAseq dataset.

## Materials and Methods

### Collection of Patient Samples

Ninety-seven cases of ovarian carcinoma treated between 1999 and 2009 were identified from the archive files of the Department of Pathology, ASST Spedali Civili of Brescia (Brescia, Italy) and included in the study. Hematoxylin & Eosin (H&E) stained slides were reviewed by an expert (LA) for appropriate classification according to the WHO 4th Edition (2014). All patients were treated and followed at the Division of Obstetrics and Gynecology ASST Spedali Civili di Brescia, Brescia, Italy. Clinical and pathology data are summarized in **Table 1** and the full dataset in **Supplementary Table S1**. The study was approved by the local IRB (WW-IMMUNOCANCERhum, NP-906, NP-1284).

**Table 1:** Clinicopathological features of the entire cohort of patients.

Patient characteristics		All	HGSC	CCC	EC
		n. (%)	n. (%)	n. (%)	n. (%)
**All**		97 (100)	59 (61)	18 (19)	20 (21)
**Age**					
** **	median (IQR)	58 (49–69)	61 (49–70)	56 (49–68.25)	53.5 (46–60)
**Menopause**					
	no	31 (32)	16 (27)	6 (33)	9 (45)
	yes	64 (66)	41 (69)	12 (67)	11 (55)
	NA	2 (2)	2 (3)	0 (0)	0 (0)
**FIGO Stage**					
	I–II	37 (38)	7 (12)	13 (72)	17 (85)
	III–IV	60 (62)	52 (88)	5 (28)	3 (15)
**Residual tumor**					
	No	52 (54)	18 (31)	15 (83)	20 (100)
	Yes	44 (45)	41 (69)	3 (17)	0 (0)
	NA	1 (1)	0 (0)	0 (0)	0 (0)
**Peritoneal cytology**					
	negative	38 (39)	12 (20)	11 (61)	15 (75)
	positive	55 (57)	47 (80)	6 (33)	3 (15)
	NA	4 (4)	0 (0)	1 (6)	2 (10)
**Chemotherapy response**					
	not response/partial	16 (16)	12 (20)	4 (22)	0 (0)
	complete	77 (79)	46 (78)	14 (78)	17 (85)
	NA	4 (4)	1 (2)	0 (0)	3 (15)
**Platinum sensitivity**					
	resistant	15 (15)	15 (25)	0 (0)	0 (0)
	partial sensitive	16 (16)	9 (15)	6 (33)	1 (5)
	sensitive	62 (64)	34 (58)	12 (67)	16 (80)
	NA	4 (4)	1 (2)	0 (0)	3 (15)
**Platinum re-eligibility**					
	no	19 (32)	19 (32)	–	–
	yes	39 (66)	39 (66)	–	–
	NA	1 (2)	1 (2)	–	–
**CD3 (cells/mm^2^)**					
	μ_g_	97	130	42	86
**CD163 (cells/mm^2^)**					
	μ_g_	365	446	285	253

HGSC, High-Grade Serous Carcinoma; CCC, Clear Cell Carcinoma; EC, Endometrioid Carcinoma; IQR, interquartile range; NA, not available; μ_g,_ geometric mean.

### Immunohistochemistry

Immunohistochemistry was performed on four-micron FFPE tissue sections with anti-CD3 (clone LN10, 1:100) anti-CD163 (clone 10D6, 1:50, Thermo Scientific) and anti-CD303/BDCA2 (clone 124B3.13, 1:75, Dendritics) antibodies, recognizing respectively T-cells, TAMs and plasmacytoid dendritic cells (PDCs). CD3 and CD163 stains were performed on Bond Max automatic immunostainer (Leica Biosystems). Immunostains for anti-CSF-1R (clone FER216, 1:1,500, Millipore), anti-IRF-1 (clone D5E4, 1:100, Cell Signaling), anti-phospho-STAT1 (clone Tyr701 rabbit, 1:500, Cell Signaling), TREM2 (anti-TREM2 antibody (clone D8I4C, 1:100, Cell Signaling Technology) and Granzyme-B (anti-GZMB antibody, clone GrB-7, 1:20, Dako) were performed manually upon microwave or thermostat bath oven epitope retrieval in ethylenediamine tetra-acetic acid (EDTA) buffer (pH 8.00). Immunoreaction was revealed by using Novolink Polymer (Leica Microsistem) followed diaminobenzidine as chromogen and with hematoxylin as nuclear counterstain. For double immunostain, after completing the first immune reaction, the second was visualized using Mach 4 MR-AP (Biocare Medical), followed by Ferangie Blue.

### RNAscope

To localize CXCL10 positive cells, tissues were analyzed with RNAscope assay (Advanced Cell Diagnostics, Newark, CA, USA) using RNAscope 2.5 HD Assay-RED kit and Hs-CXCL10-C2 probe (Cat No. 311851-C2) recognizing the nt 2 to 1,115 of the CXCL10 reference sequence NM_001565. The sections from fixed human tissue blocks were treated following the manufacturer’s instructions. Briefly, freshly cut 3 μm sections were deparaffinized in xylene and treated with the peroxidase block solution for 10 min at room temperature followed by the retrieval solution for 15 min at 98°C and by protease plus at 40°C for 30 min. Control probes included Hs-POLR2a-C2 (Cat No. 310451) and DapB-C2 (Cat No. 310043-C2). The hybridization was performed for 2 h at 40°C. The signal was revealed using RNAscope 2.5 HD Detection Reagent and FAST RED. Combined RNAscope and immunohistochemistry (for CD163, IRF1, Phospho-STAT1, CSFR1 and TREM2) were used to identify the cellular source of CXCL10. To this end, CXCL10 detection by RNAscope was followed by immunoreaction was visualized using Novolink Polymer (Leica Microsistem) followed by DAB or using Mach 4 MR-AP (Biocare Medical) followed by Ferangi Blue (Biocare Medical).

### Digital Image Analysis

Cell density of selected immune populations was analyzed using digital microscopy. The absolute cell count was quantified automatically using a custom-programmed script in Cognition Network Language based on the Definiens Cognition Network Technology platform (Definiens AG, Munich, Germany). Briefly, CD3, CD163, and BDCA2 stained slides were digitalized using an Aperio ScanScope CS Slide Scanner (Aperio Technologies, (Leica Biosystem, New Castle Ltd, UK) at 40× magnification and analyzed using Tissue Studio 2.0 (Definiens AG). The quantitative scoring algorithm was customized using commercially available templates (**Supplementary Figure S1**). The image analysis pipeline comprised segmentation of nucleus objects and cell classification based on a pre-trained decision tree, according to staining intensity. Immune cell counts were expressed as the number of positive cells/mm^2^ of ovarian cancer area.

### RNA Extraction and Gene Expression Analysis

A custom immune signature of 105 genes, selected on the basis of a PubMed literature search, was devised for the digital transcript counting, including targets for innate and adaptive immunity, co-stimulatory or immune effector molecules, and chemokines with their corresponding receptors (**Supplementary Table S2**). A representative formalin-fixed and paraffin-embedded (FFPE) tumor block was retrieved from the biobank. Tissue was cut into 10/20 μm sections and treated with Deparaffinization Solution (QIAGEN). RNA was extracted using Qiagen RNeasy FFPE kit (QIAGEN). Total RNA concentration and proteins contamination were determined by a Nanodrop spectrophotometer (Nanodrop Technologies, Ambion, Waltham, MA, USA). Quality of RNA was monitored using Agilent 2100 Bioanalyser System (AGILENT). Total RNA (100 ng) was assayed on a nCounter platform using NanoString technology (NanoString, Seattle, WA), testing the whole set of 105 endogenous genes, five housekeeping genes, six ERCC (External RNA Control Consortium) positive controls and eight ERCC negative controls (**Supplementary Table S2**). Raw mRNA counts were normalized applying a sample-specific correction factor to all the target probes per manufacturer’s recommendations (technical normalization with Positive Controls Normalization spiked in every assay and biological normalization using housekeeping genes). The resulting normalized counts were used in downstream analyses. Pearson correlation analysis between log2 IHC densities and log2 mRNA Nanostring normalized counts were performed by Hommel correction for multiple comparisons deriving the OC-IS^30^ Immune signature. A penalized linear ridge regression was applied to the Z-scores OC-IS^30^ gene expression to weight the signature for its application in external datasets. For the Nanostring data heatmaps, the above values were turned into Z-scores. For Nanostring gene expression analysis, normalization and differential expression (DE) were performed with Nanostring nCounter nSolverTM 4.0 (Nanostring MAN-C0019-08), with Nanostring Advanced Analysis Module 2.0 plugin (Nanostring MAN-10,030–03) following the Nanostring Gene Expression Data Analysis Guidelines (Nanostring MAN-C0011-04).

### External Cohort Validation (TCGA)

For external cohort *in-silico* analysis, publicly available HGSC data from The Cancer Genome Atlas [TCGA-OV, N = 312 (6)] have been considered. Records of cases with full annotation on tumor stage, survival data, mutational status of BRCA1 and BRCA2 genes (both germline and somatic) and tumor mutational burden (TMB) were retrieved through the Computational Biology Center Portal (cBio): http://www.cbioportal.org/ and downloaded on 15th Feb 2020. Data of mRNA expression profile (TCGA_eset) were downloaded through the curated OvarianData v.1.24.0 R package (16)). The TCGA dataset was investigated computing the OC-IS^30^ Immune signature and the whole immune fraction applying CIBERSORTx (17) using a signature matrix (18) able to compute the global immune transcriptome. Raw counts for primary solid tumor samples of further eight TCGA projects (TCGA-BLCA, TCGA-BRCA, TCGA-COAD, TCGA-HNSC, TCGA-LUAD, TCGA-LUSC, TCGA-SKCM, and TCGA-UCEC) were downloaded from GDC Legacy Archive (hg19) using TCGAbiolinks R/Bioconductor package. The FFPE samples were removed. Normalized expression levels, by upper quartile normalization measured in RSEM were obtained. Overall stage was included as clinical variables and 4,496 cases, including the OV dataset, were available for the analysis.

### Generation of Human Monocyte-Derived Mϕ

PBMCs were obtained from buffy coats of healthy volunteer blood donors (courtesy of the Centro Trasfusionale, ASST Spedali Civili, Brescia) by Ficoll–Paque (GE Healthcare, Milano, Italy) density gradient centrifugation at 360×*g* for 30 min. Peripheral blood CD14^+^ monocytes were magnetically sorted with human Pan Monocyte Isolation Kit (Cat. No. 130-096-537, Miltenyi Biotec, Bergisch Gladbach, Germany) following manufacturer’s instructions. Isolated monocytes (7 × 10^5^ cells/ml) were seeded in RPMI 1640 medium (Cat. No. 1-41-F01-I Bioconcept, Allschwil, Swiss) supplemented with 10% FBS (Biochrom GmbH, Berlin, Germany), GlutaMAX™-I (Cat. No. 35050-038, Life Technologies, Carlsbad, CA), 20 U/ml penicillin, 20 µg/ml streptomycin (Cat. No. 15070-063, Life Technologies, Carlsbad, CA). After overnight culture, non-adherent cells were removed by washing with DPBS (Cat. No. 14190-094, Life Technologies, Carlsbad, CA) and adherent monocytes were cultured over 7 days in the presence of 100 ng/ml human M-CSF premium grade (Cat. No. 130-096-489, Miltenyi Biotec) to generate macrophages (M0). The medium was not replaced throughout the culture period. Macrophages polarization was obtained by replacing culture medium with fresh RPMI 1640 medium supplemented with 10% FBS and containing 50 ng/ml recombinant human IFN-γ (Cat. No. 300-02, Peprotech, London, UK) or 20 ng/ml recombinant human IFN-γ (Peprotech) + 100 ng/ml LPS from *Escherichia coli* O55:B5 (Cat. No. L6529 Sigma-Aldrich, St. Louis, MO) (for M1 polarization) or 20 ng/ml Recombinant human IL-4 premium-grade (Cat. No. 130-093-920, Miltenyi Biotec) or 20 ng/ml Recombinant human IL-10 research-grade (Cat. No. 130-093-948, Miltenyi Biotec) (for M2 polarization) for 4 or 18 h. M0 cultured with fresh medium without polarization cytokines was used as control.

### Cell-Block Preparation

Cell suspensions of macrophages were centrifuged for 10 min at 3,000 rpm. A solution of plasma (100 μl, kindly provided by Centro Trasfusionale, ASST Spedali Civili, Brescia) and HemosIL8 RecombiPlasTin 2G (200 μl, Instrumentation Laboratory, Bedford Ma, USA, Cat. No. 0020003050) (1:2) were added to cell pellets, mixed until the formation of a clot, then placed into a labeled cassette. The specimen was fixed in 10% formalin for 1 h followed by paraffin inclusion.

### qRT-PCR

IL-6, CXCL10 and COX2 mRNA targets were quantified by reverse transcription-polymerase chain reaction (qRT-PCR) assay using the Vii-A 7 Real-Time PCR System (Applied Biosystems, Thermo Fisher Scientific, Waltham, MA, USA). Total RNA was purified from M0, M1 and M2 macrophages using the RNeasy^®^ Mini Kit (Cat. No. 74104, Qiagen). The cDNA was synthesized by iScript gDNA cDNA Synthesis kit (Cat. No. 1725035, Bio-Rad Laboratories Inc., Hercules, CA, U.S.A.) from 150 ng of total RNA, in a total volume of 20 μl. About 1 μl of the cDNA synthesis reaction was used for the specific amplification of the target transcripts. The Ribosomal Protein S18 (RPS18) transcript was used as normalization control. The qPCR was performed in a total volume of 10 μl with TaqMan^®^ Universal Master Mix II (Cat. No. 4369016, Applied Biosystems, Thermo Fisher Scientific) and the Gene Expression Assay (**Supplementary Table S3**). The threshold cycle (Ct) was determined for each sample and quantification was performed using the comparative Ct method. ΔCt was derived as Ct_Target_ − Ct_Housekeeping_ and considered for statistical analysis.

### Western Blotting

The intracellular levels of targets and actin proteins were determined by western blotting. Cells were washed, re-suspended in RIPA lysis buffer (Cat. No. 89900, Pierce, Thermo Fischer Scientific) with a Protease Inhibitor Cocktail (Cat. No. 78440, Sigma-Aldrich) and sodium orthovanadate (Na_3_VO_4_) (Cat. No. 450243, Sigma-Aldrich), and kept in ice for 10 min. After 20 min centrifugation at 12,000×*g* at 4°C, the supernatant was collected and protein concentration determined by Bradford assay. A total of 20 μg of proteins was loaded on 4–12% NuPAGE^®^ Bis-Tris Mini Gels (Cat. No. NP0335, Invitrogen™, Thermo Fisher Scientific) under reducing condition and transferred onto a PVDF membrane (Cat. No. LC2007, Invitrogen™, Thermo Fisher Scientific). Membranes were incubated in the blocking solution 5% BSA (Cat. No. A3059, Sigma-Aldrich) in T-TBS (TBS, 0.05% Tween 20) (Cat. No. 28360, Invitrogen™, Thermo Fisher Scientific) for 1 h at room temperature; subsequently membranes were exposed to primary antibodies diluted in blocking solution, for 16 h at 4°C. Primary antibodies are listed in **Supplementary Table S4**. After washing in TBS-T, the blots were incubated with the appropriate secondary antibody (anti-Rabbit Cat. No. sc-2077 *Santa Cruz* Biotechnology, Inc., Dallas, TX, USA), conjugated with horseradish peroxidase for 1 h at room temperature. Immunoreactive proteins were detected by SuperSignal™ West Pico Chemiluminescent Substrate (Cat. No. 34577, Thermo Fisher Scientific) and visualized by autoradiography.

### Statistical Analysis

For histological, clinical, and pathological analysis the qualitative variables were described as absolute and relative frequencies. We considered overall survival (OS) and progression-free survival (PFS). In the absence of any events, survivals were censored at last follow-up visit. Qualitative variables were compared between groups using Chi-square test, quantitative one by t-test, Mann–Whitney test or ANOVA, and post-hoc pairwise comparisons as appropriate. By evaluation of Q–Q plots and applying the Shapiro–Wilk Test immune cells densities’ distribution followed a log-normal distribution; for statistical analysis log_2_ values of densities were used. Median values of continuous variables’ distributions were set as cut-offs for dichotomization. Univariable and multivariable analyses were performed with Cox proportional hazard models. For all analyses the proportional hazards assumption was tested and verified; estimates were reported as hazard ratio (H.R) with 95% Confidence Intervals (CI). In all analyses a two-tailed P value <0.05 was considered significant. GraphPad Prism (San Diego, CA, USA), and R (version 3.6.2) were used for statistical analysis.

### scRNAseq Data Analysis

Processing of the Pan-Cancer Blueprint dataset. We downloaded the raw datasets and selected the myeloid cells dataset (using the article annotation with the mention “Myeloid” in the cell type metadata, 37,334 cells) of Qian et al. (19) from a web server (http://blueprint.lambrechtslab.org). Cells were merged using the Canonical Correlation Analysis (CCA) and the Mutual Nearest Neighbors (MNN) algorithms and we selected the 5,000 most variable genes (following the Seurat 3 pipeline). We next performed Louvain graph-based clustering. At the resolution 0.6 we obtained 27 clusters. Eleven clusters (c1, 2, 3, 4, 5, 9, 10, 12, 18, 19, 26) expressed high levels of CD68 and were labeled as macrophages.

## Results

### Heterogeneity of T-Cells and TAMs Immune Contexture in OC Subtypes

**Figure 1:**
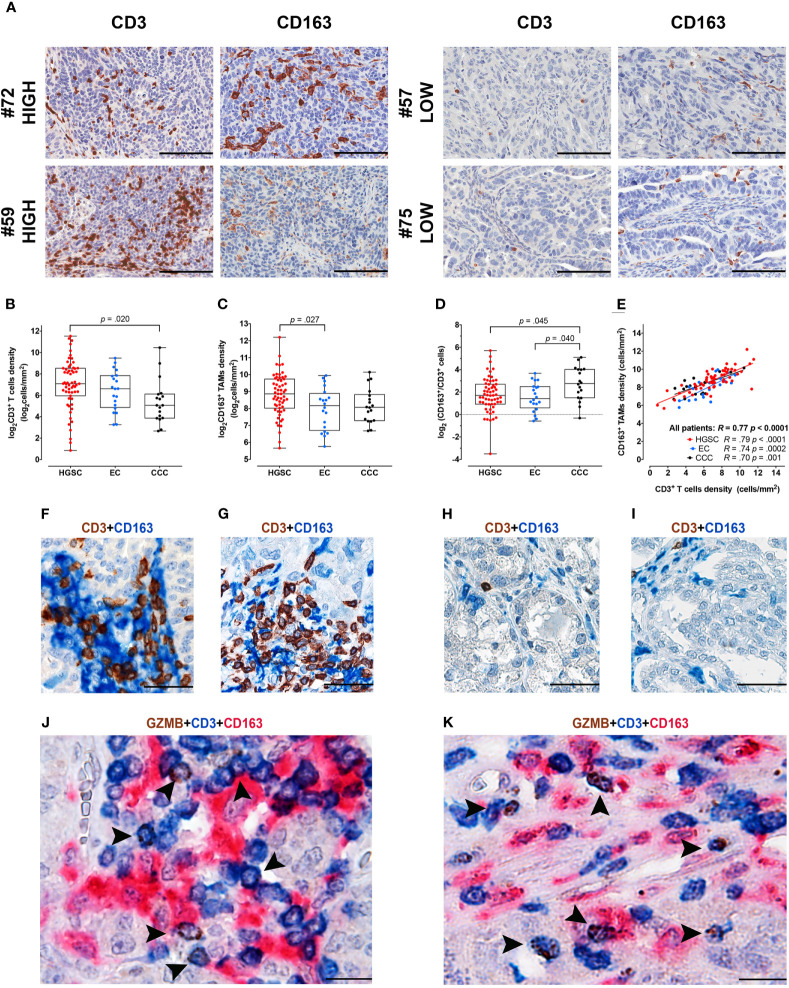
Immune contexture in OCs by digital microscopy and interactions of CD3^+^ T-cells and CD163^+^ TAMs. **(A)** Sections are from four representative HGSCs (case #72, #59, #57 and #75) and immunostained for CD3 and CD163, recognizing T-cells (left column) and tumor-associated macrophages (TAMs) (right column). Case #72 and #59 are HGSCs with rich immune cell density, characterized by high CD3^+^ T-cells and CD163^+^ TAMs, intraepithelial and stromal infiltrates. In opposition case #57 and #75 correspond to two HGSCs with low immune cell density, showing very low-number of CD3^+^ T-cells and CD163^+^ TAMs. Sections are counterstained with hematoxylin. Images had been acquired form Digitalized slides using Aperio Image Scope (Leica Biosystems) Magnification 200×; scale bar 100 um. Box plots showing CD3^+^ T-cells **(B)** and CD163^+^ TAMs **(C)** densities in different OCs subtypes. Box-plots showing the ratio of CD163^+^/CD3^+^ densities **(D)**. Scatter plot **(E)** illustrating the correlation analysis between CD163^+^ and CD3^+^ immune cells’ densities in the whole cohort and among different OCs subtypes. P values estimated by One-way ANOVA with Tukey’s correction for multiple comparisons in **(B–D)**; R and P values estimated by Pearson correlation test in **(E)**. Sections from HGSCs **(F, G, J, K)** and CCCs **(H, I)** cases and immunostained as labeled, showing common interactions between T-cells and CD163^+^ TAMs observed in HGSCs and not in CCCs; magnification: 400× **(F–I)**, 600× **(J, K)**; scale bar: 50 um **(F–I)**; 33 um **(J, K)**. HGSC, High Grade Serous Carcinoma; EC, Endometrioid Carcinoma; CCC, Clear Cell Carcinoma.

By digital image analysis on stained sections, we measured T-cells and Mϕ immune-contexture in a retrospective cohort of OC (n = 97) and explored associations (**Figures 1A–I**). To this end, serial sections from a representative tumor area of primary OC obtained from a single tissue block were stained for CD3 and CD163. The density of T-cells resulted extremely variable ranging from 2 to 2,967 cells/mm^2^ (mean 283 cells/mm^2^, median 106 cells/mm^2^, IQR 34–311 cells/mm^2^); similarly, CD163^+^ TAMs counts varied from 51 to 4,714 cells/mm^2^ (mean 529 cells/mm^2^, median 372 cells/mm^2^, IQR 224–704 cells/mm^2^). The full dataset is reported in **Supplementary Table S1**. Both densities’ distribution followed a log-normal distribution, log_2_ values of densities were thus used for statistical analysis (**Supplementary Figure S2**). Subgroup analysis among OCs with different histology indicates that HGSCs are significantly more infiltrated by CD3 T-cells, compared to CCCs (p = .02, **Figure 1B**) and by CD163^+^ TAMs, compared to ECs (p = .027, **Figure 1C**). Moreover, the TAMs/T-cells ratio resulted significantly higher in the CCC subtype compared to HGSC (p = .045) or to EC subtypes (p = .04, **Figure 1D**). Both immune populations resulted highly correlated (R = .77, p=<.0001) also when considering the OC subgroups HGSC (R=.79, p<.0001), CCC (R=.74, p = .0002) and EC (R = .70, p = .001) respectively (**Figure 1E**). As indicated by double and triple stain for CD3, GZMB and CD163, T-cells/TAM interactions were commonly observed in HGSCs (**Figures 1F, G, J, K**) but not in CCCs (**Figures 1H, I**). This analysis highlights differences in immune contexture between OCs subtypes, with immune infiltrated HGSC and T-cell poor CCC positioned at the extreme of a functional spectrum.

### T-Cells and TAMs Immune-Contexture Predict Outcome in HGSC

**Figure 2:**
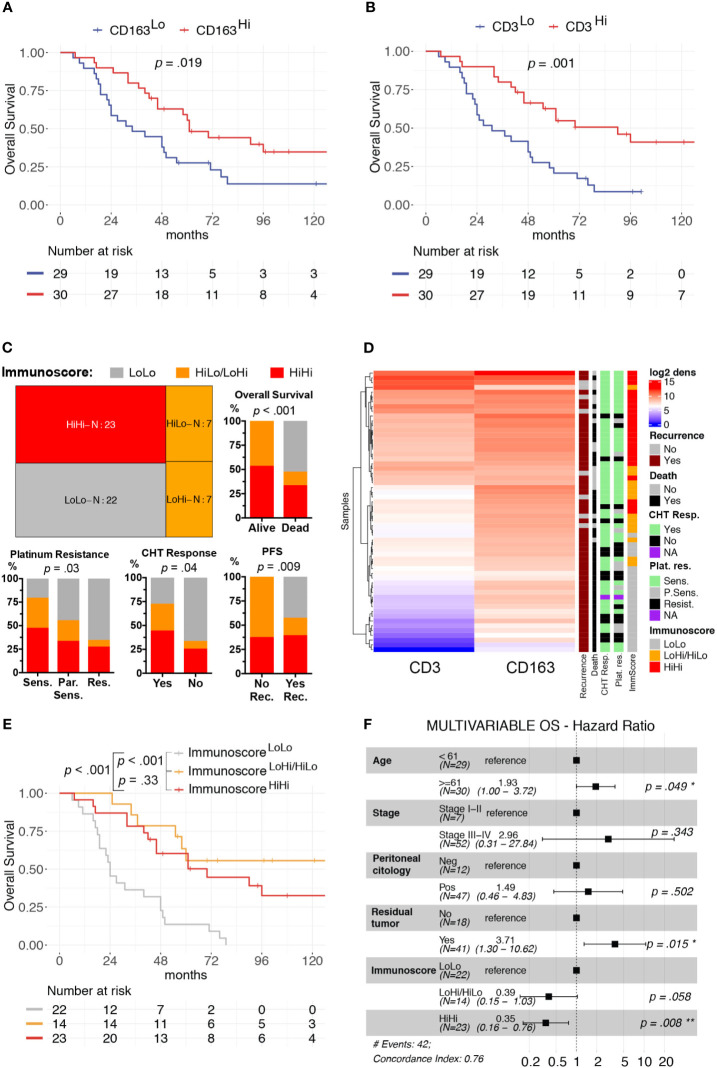
Clinical significance of CD3^+^ T-cells and CD163^+^ TAMs density in HGSC. Univariable overall survival estimates (Kaplan–Meier method) according to CD3^+^ T-cells **(A)** and CD163^+^ TAMs **(B)** densities; p-values estimated with log-rank test. Treemap showing subgroup composition based on immunoscore and results of association analysis between immunoscore and chemotherapy response (CHT resp.), platinum resistance (Plat. Res.) and survival events (Death or Recurrence) **(C)**. Heatmap of unsupervised hierarchical clustering by Euclidean distance of log_2_CD3^+^ T-cells and log_2_CD163^+^ TAMs densities, each row represents a patient **(D)**. Univariable overall survival estimate (Kaplan–Meier method) of immunoscore classes **(E)**, pairwise comparisons p-values adjusted with FDR. Forest-plot of multivariable overall survival analysis **(F)**. Cut-offs for CD3^Hi^ and CD163^Hi^ densities were set at median values for each distribution. *P < 0.05; **P < 0.01.

We focused our clinical correlation analysis on HGSCs, the more represented OC subtype (**Table 1**). The mean log_2_ CD3^+^ T-cells density was significantly higher in patients with low-risk features, such as Stages I–II (p = 0.03) and negative peritoneal cytology (p = 0.016). Moreover, a higher immune cells infiltrate was associated with a better response to first-line treatment. Specifically, a complete response to chemotherapy was associated with higher CD3^+^ T-cells density (p = 0.04). Moreover, platinum sensitivity and platinum re-eligibility were associated either with higher CD3^+^ T-cell density (p = 0.008, p = 0.009) and CD163^+^ TAM density (p = 0.028, p = 0.031). We further expanded this analysis by evaluating the relevance of the immune contexture in terms of clinical outcome. To this end, subgroups were defined using the median values of each immune cell densities’ distributions as cut-offs (CD3^Hi^ vs CD3^Low^ and CD163^Hi^ vs CD163^Lo^). The univariate survival analysis, reported in **Supplementary Table S5**, confirmed, both for OS and PFS respectively, the association with worse prognosis of well-known clinical variables as higher Stages III–IV (H.R. 10.56, p = .02; H.R. 6.63, p = .009), macroscopic residual tumor (H.R. 7.15, p <.001; H.R. 2.89, p = .002), and positive peritoneal cytology (H.R. 4.25, p <.007; H.R. 4.41, p = .002). The CD163^Hi^ group experienced a better OS (H.R. 0.48, p = .019, **Figure 2A**) and better PFS (H.R. 0.56, p = .042, **Supplementary Figure S3A**); besides, the CD3^Hi^ had a better OS (H.R. 0.35, p = .001, **Figure 2B** and **Supplementary Figure S3B**).

As both T-cells and TAMs were associated with favorable prognosis and were positively correlated, we further expanded our analysis by identifying three additional groups, namely the Immunoscore^LoLo^ (CD3^Lo^ and CD163^Lo^), the Immunoscore^HiLo/LoHi^ (CD3^Hi^ and CD163^Lo^, or CD3^Lo^ and CD163^Hi^) and the Immunoscore^HiHi^ (CD3^Hi^ and CD163^Hi^) groups, as shown in the Treemap (**Figure 2C**). A higher Immunoscore was associated with both chemotherapy response (p = 0.04) and platinum sensitivity (p = 0.03, **Figures 2C, D**). In addition, univariate survival analysis showed a better OS (Immunoscore^HiLo/LoHi^: H.R. 0.17, p <0.001; Immunoscore^HiHi^ H.R. 0.28, p <0.001; **Figure 2E**) and PFS (Immunoscore^HiLo/LoHi^: H.R. 0.23, p <0.001; Immunoscore^HiHi^ H.R. 0.41, p = 0.005; **Supplementary Figure S3C** and **Supplementary Table S5**) for higher immunoscore compared to Immunoscore^LoLo^ as reference group. In term of OS, the multivariable survival analysis (**Figure 2F**) confirmed a favorable prognosis for Immunoscore^HiHi^ (H.R. 0.35, p = 0.008) compared to Immunoscore^LoLo^ OCs; moreover, a better PFS was observed for Immunoscore^HiLo/LoHi^ (H.R. 0.40, p = 0.031) and Immunoscore^HiHi^ (H.R. 0.49, p = .037) groups compared to Immunoscore^LoLo^ OCs (**Supplementary Figure S3D**). The immunoscore variable was even more relevant than others clinical covariates as Stage or positive peritoneal cytology that lost the statistical significance in the multivariable model.

### OC-IS^30^ Immune Signature Marks Immune Infiltrated OCs

**Figure 3:**
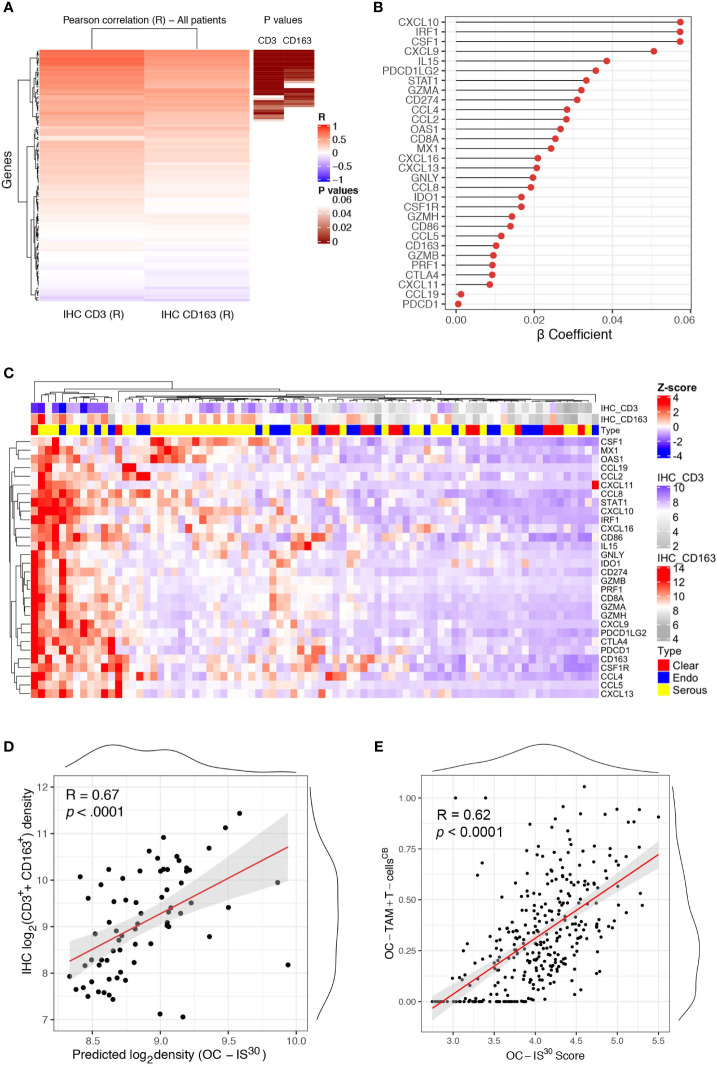
OC-IS^30^ identify inflamed OCs. Heatmap of Pearson R coefficients of correlation analysis between log_2_Immune cells densities (for CD3^+^ T-cells and CD163^+^ TAMs) and log_2_normalized Nanostring gene signature. The 30 significant correlated genes (OC-IS^30^ signature) are underlined in brown (Hommel adjusted p-values) **(A)**. Lollipop chart showing β coefficients of the penalized ridge linear regression, in descending order based on the weight of each gene, for the prediction of the sum of CD3^+^ and CD163^+^ density in the OCs cohort **(B)**. Heatmap of Z-score of OC-IS^30^ signature in the OCs training cohort; top annotations showing histology subtype and CD3^+^ T-cells and CD163^+^ TAMs densities **(C)**. Scatter plot of the predicted OC-IS^30^ density against the observed CD3^+^ T-cell and CD163^+^ TAMs density in the training cohort (R = 0.67, p < 0.0001) **(D)**. Scatter plot of the OC-IS^30^ score against the sum of T-cell and TAMs fractions estimated by CIBERSORTx in the TCGA cohort (R = 0.62, p < 0.0001) **(E)**.

We further expanded our findings by measuring the expression of a custom immune signature in the OC cohort using Nanostring technology. The custom immune signature included one-hundred and five targets covering genes relevant for innate and adaptive immunity, effector molecules, and chemokine with their corresponding receptors (**Supplementary Table S2**). Eighty-one cases were deemed suitable for Nanostring-based gene expression analysis (GEA). A set of healthy ovarian tissue (n = 12) was included as control group. Differential expression analysis revealed a significant up-regulation (adj. p-values <0.05) of a large set of targets in OCs compared to controls (**Supplementary Figure S4A**). A supervised analysis based on histology subgroups revealed lack of significant differences for most of the targets (**Supplementary Figure S4B**), with the exception of four targets including CSF1, the latter significantly higher in HGSC and correlating with a high density of CD163^+^ TAMs (**Supplementary Table S6**). To extend the finding obtained by digital microscopy analysis, we correlated the GEA of OCs cases with the corresponding T-cell and TAMs density. Of technical relevance, among all 105 genes of the tested signature, none was inversely correlated with immune cells densities (**Figure 3A**). In addition, a set of thirty genes (from here referred as OC-IS^30^) (**Supplementary Table S6**) showed a significant direct positive correlation with CD3^+^ T-cells or CD163^+^ TAMs tissue densities (adj. p-value <0.05) (**Figures 3B–D**). This finding was confirmed and extended by applying CIBERSORTx (17) to the external OV-TCGA dataset using a signature matrix (18) able to compute T-cells and macrophages (**Figure 3E**). Of note, the OC-IS^30^ gene set contained targets relevant to T-cell attracting chemokines (CXCL10, CXCL9, CXCL11, and CXCL16), immune effector function (GZMA, GNLY, PRF1, GZMB, GZMH), Mϕ biology (CSF1, CSF1R, CCL2, CCL4, and CD163), immune checkpoints (IDO1, CTLA4, CD274, PDCD1LG2, and PDCD1) and interferon signature (CXCL10, CXCL9, CXCL11, CXCL16, CD274, IDO1, STAT1, MX1, OAS1). The latter finding suggest an ongoing interferon response in immune infiltrated OCs; based on very low density of PDCs infiltration in primary OC (**Supplementary Figure S5**), our data are more consistent with an IFNγ signature.

### OC-IS30 Predicts Favorable Outcome in HGSC and Across Human Cancer Types

**Figure 4:**
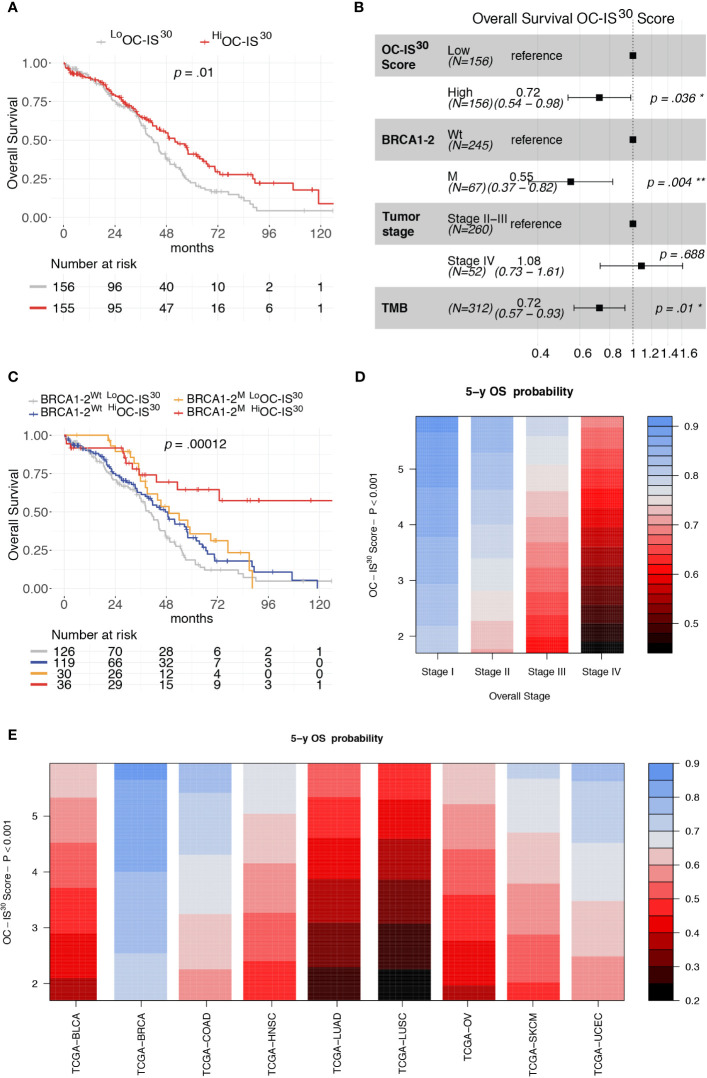
Prognostic significance of OC-IS^30^ in the TCGA datasets. Prognostic significance of ^Hi^OC-IS^30^ group by univariable **(A)** and multivariable **(B)** overall survival analysis; Kaplan–Meier curves of the OC-IS^30^ status combined with BRCA1 and BRCA2 mutational status **(C)**. Contour plots from a multivariable Cox model analyzing samples from 9 TCGA datasets (N = 4,496) including the Overall Stage, the OC-IS^30^ Score [weighted log(normalized gene expression+1) applying the coefficients defining the OC-IS^30^ signature (**Figure 3B**)] and the tumor site showing the significant, independent and additive favorable prognostic significance (color gradient) of lower Stage **(D)** and higher OC-IS^30^ score **(E)** across the different tumor sites. P values estimated by log-rank test in **(C)** and by Cox models in **(A, B, D, E)**. BLCA, Bladder Urothelial Carcinoma; BRCA, Breast invasive carcinoma; COAD, Colon adenocarcinoma; HNSC, Head and Neck squamous cell carcinoma; LUAD, Lung adenocarcinoma; LUSC, Lung squamous cell carcinoma; OV, Ovarian serous high grade carcinoma; SKCM, Skin Cutaneous Melanoma; UCEC, Uterine Corpus Endometrial Carcinoma. *P < 0.05; **P < 0.01.

The clinical significance of OC-IS^30^ was tested in the external OV-TCGA dataset (6) containing 312 HGSCs annotated in term of clinical and molecular finding (Stage, Overall Survival, mutational status of *BRCA1* and *BRCA2* genes, and tumor mutational burden (TMB). OC-IS^30^ expression was not significantly associated with tumor stage (p = 0.09), BRCA1-2 mutations (p = 0.098) or TMB (p = 0.08), as reported in **Supplementary Figures S6A–C**. For the distribution of OC-IS^30^ score the median value was set as cut-off point for identification of rich (^Hi^) or poor (^Lo^) immune represented group. The ^Hi^OC-IS^30^ group was associated with a better OS at univariable analysis (H.R. 0.68, CI_95%_ 0.50–0.91, p = 0.01, **Figure 4A**), as well as using a multivariable model including well known prognosticators (**Figure 4B**). Specifically, the multivariable analysis confirmed the favorable prognostic significance of OC-IS^30-Hi^ (H.R 0.72, p = 0.036) independent from BRCA1-2 mutations (H.R. 0.55, p = 0.004) and TMB (H.R. 0.72, p = 0.01); the positive combined effect of ^Hi^OC-IS^30^ and BRCA1-2 mutations is reported by Kaplan–Meier curves in **Figure 4C**. By exploring the TCGA datasets, we expanded our analysis across different cancers and found that the OC-IS^30^ predict favorable outcome independent from Overall Stage and cancer site (H.R. 0.85, CI_95%_ 0.79–0.91, p <0.0001, **Figures 4D, E**).

### M1-Polarized TAMs Hallmark Immune-Infiltrated HGSC But Not T-Cell Poor CCC

Data from the literature (20) and from this study using OC-IS^30^ indicate a clinical benefit of the IFNγ response in OCs. The observed effect might derive from an IFNγ response by tumor cells or host immune cells, particularly TAM. To answer this question at the single-cell level we tested the expression and cellular localization of a set of M1- and M2-type macrophages (Mϕ) markers including IRF1, IRF4, CD163, and pSTAT1Y701 by immunohistochemistry. To validate these markers for formalin-fixed cells, we initially monitored their expression and cellular localization on cell-block sections of monocyte-derived macrophages. To this end, we generated monocyte-derived (M0) Mϕ and polarized them to M1-type (M1^IFN^γ and M1^IFN^γ^+LPS^) and M2-type (M2^IL-4^ and M2^IL-10^) Mϕ , as also confirmed by the expression of IL6 and COX2 (**Supplementary Figure S7A, B**). We found that IRF1 and pSTAT1Y701 induction and nuclear localization were strictly coupled with M1 polarization, being limited (IRF1) or totally absent (pSTAT1Y701) in M2^IL-4^ and M2^IL-10^ Mϕ (**Supplementary Figures S7C, D**). On the contrary, IRF4 results strongly modulated in M1^IFN^γ^+LPS^ and M2^IL-4^ Mϕ with a basal level of nuclear expression also in M1^IFN^γ Mϕ (**Supplementary Figure S7E**). CD163 is induced in Mϕ generated by IL-10- and CSF1, as measured by flow cytometry (21), and for this reason it has been considered an M2-type Mϕ marker. We found that its cytoplasmic expression is, however, easily detectable by IHC in all polarization conditions (M0, M1, and M2) (**Supplementary Figure S7C**), suggesting that CD163 expression is promiscuous in Mϕ populations and cannot be used as M2-specific marker by IHC.

**Figure 5:**
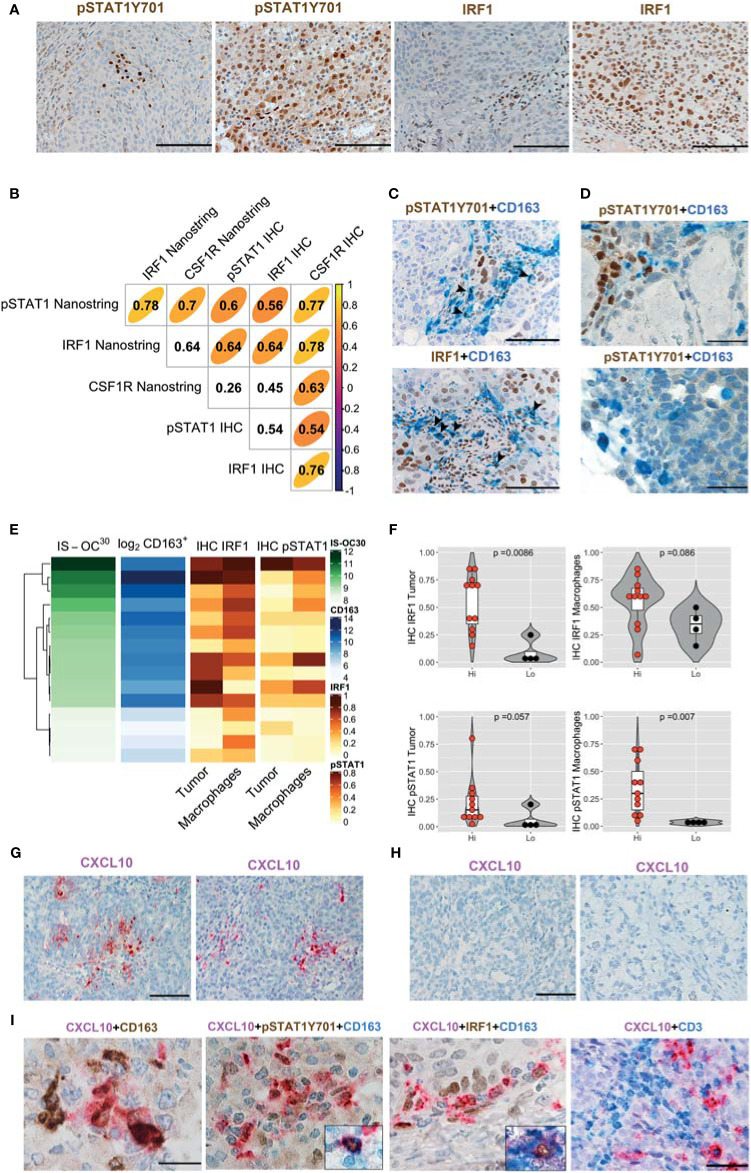
IFNγ polarization on cancer cells and stromal M1 type macrophages (Mϕ) in OCs. Sections from OCs cases and immunostained as labeled **(A, C, D)**. Magnification 200× (**A, C**; scale bar 100 um); magnification 400× (**D**, scale bar 50 um). Different levels of pSTAT1Y701 and IRF1 expression in ^Lo^OC-IS^30^CD163^L^ and ^Hi^OC-IS^30^CD163^Hi^ HGSCs cases **(A)**. Spearman correlogram of IRF1, pSTAT1Y701 and CSF1R in OCs by IHC and Nanostring **(B)**. A fraction of HGSCs infiltrating CD163^+^ TAMs expresses pSTAT1Y701 and IRF1 **(C)**. Sections of CCCs showing that this tumor histotype is largely devoid of pSTAT1Y701^+^ TAMs **(D)**. Heatmap showing the IHC IRF1 and pSTAT1Y701 expression on different tissue compartments compared to the matched OC-IS^30^ score and log_2_CD163^+^ density **(E)**; Violin plots reporting the IHC IRF1 and pSTAT1Y701 expression in the ^Hi^OC-IS^30^CD163^Hi^ and ^Lo^OC-IS^30^CD163^Lo^ groups, p values estimated by Mann–Whitney test **(F)**. **(G–I)** Sections from OCs immunostained or subjected to *in situ* hybridization as labeled; while CXCL10 is detected in ^Hi^OC-IS^30^CD163^Hi^ HGSCs **(G)** cases it is absent in a ^Lo^OC-IS^30^CD163^Lo^ CCCs cases **(H)**. A fraction of CXCL10^+^ cells is confirmed to have a M1 Mϕ identity and areas containing CXCL10^+^ macrophages are enriched of T-cells **(I)**. Magnification: **(G, H)** 200× (scale bar 100 um) and **(I)** 600× (scale bar 33 um, first three panels) and 400× (scale bar 50 um, right panel). **(C, D)**: arrowheads pointing double positive cells.

We subsequently analyze ^Hi^OC-IS^30^CD163^Hi^ (n = 15) and ^Lo^OC-IS^30^CD163^Lo^ (n = 4) from the HGSC group. In OCs tissues, nuclear pSTAT1Y701 and IRF1 were detected in tumor cells and cells of the microenvironment (**Figure 5A**). Based on a three-tiered IHC score, we found a significant positive correlation between protein biomarkers and the corresponding mRNA level, as detected by Nanostring (**Figure 5B**). Moreover, by double stain for CD163, we could confirm nuclear reactivity for pSTAT1Y701 and IRF1 in a fraction of CD163^+^ TAMs (**Figures 5C, D**). As a relevant tissue pattern, we could detect tumor areas of “inducible” pSTAT1Y701 and IRF1 expression containing clusters of positive tumor cells and TAMs (**Figures 5A, C**). By quantitative analysis, ^Hi^OC-IS^30^CD163^Hi^ cases were significantly enriched of IRF1^+^ tumor cells (p = .0086) and pSTAT1Y701^+^ TAMs (p = .007) compared to ^Lo^OC-IS^30^CD163^Lo^ (**Figures 5E, F**). This observation suggests an M1-type polarization of CD163^+^ TAMs in immune-infiltrated OCs. We extended these findings to CCC (n = 10), a subtype displaying poor T-cells infiltration in our cohort. By double immunohistochemistry for pSTAT1Y701 and CD163, CCC resulted largely devoid on pSTAT1Y701^+^ TAMs (mean ± SD = 1.6 ± 2.0%, **Figure 5D**). These observations highlight heterogeneity in M1-type polarization in OC subtypes with diverse T-cell contexture.

### M1-Type TAMs Produce CXCL10 and Co-Localize With T-Cells

Among IFNγ targets, the chemokine CXCL10 has been shown to control T-cell recruitment into the tumor environment (22). We tested mRNA expression by using qPCR and RNAscope-based *in situ* hybridization. Both approaches demonstrate that only M1^IFN^γ and M1^IFN^γ^+LPS^ were associated with high induction of CXCL10 transcript, whereas M0, M2^IL-4^ and M2^IL-10^ Mϕ resulted largely negative (**Supplementary Figures S7F–G**). This data was confirmed by RNAscope-based *in situ* hybridization of formalin-fixed cell-block preparation (**Supplementary Figure S7F**). On biopsies, we could subsequently detect more abundant CXCL10 transcript in ^Hi^OC-IS^30^CD163^Hi^ (n = 3) cases compared to ^Lo^OC-IS^30^CD163^Lo^ (n = 3) (**Figure 5G**). Moreover, also most CCCs (n = 10) were largely devoid of CXCL10 stain (**Figure 5H**). By combining RNAscope with IHC we could confirm a M1 Mϕ identity of a fraction of CXCL10^+^ cells, in addition to CXCL10^+^ cancer cells (**Figure 5I**). The analysis of double stained sections from immune infiltrated ^Hi^OC-IS^30^CD163^Hi^ (n = 3) revealed that areas containing CXCL10^+^ macrophages are enriched of T-cells (**Figure 5I**). These findings confirmed that immune infiltrated ^Hi^OC-IS^30^CD163^Hi^ are enriched of M1-type Mϕ, producing the T-cell attracting chemokine CXCL10 and surrounded by CD3^+^ T-cells.

### A Fraction of M1-Type Mϕ in OCs Co-Expresses CSF1R and TREM2

**Figure 6:**
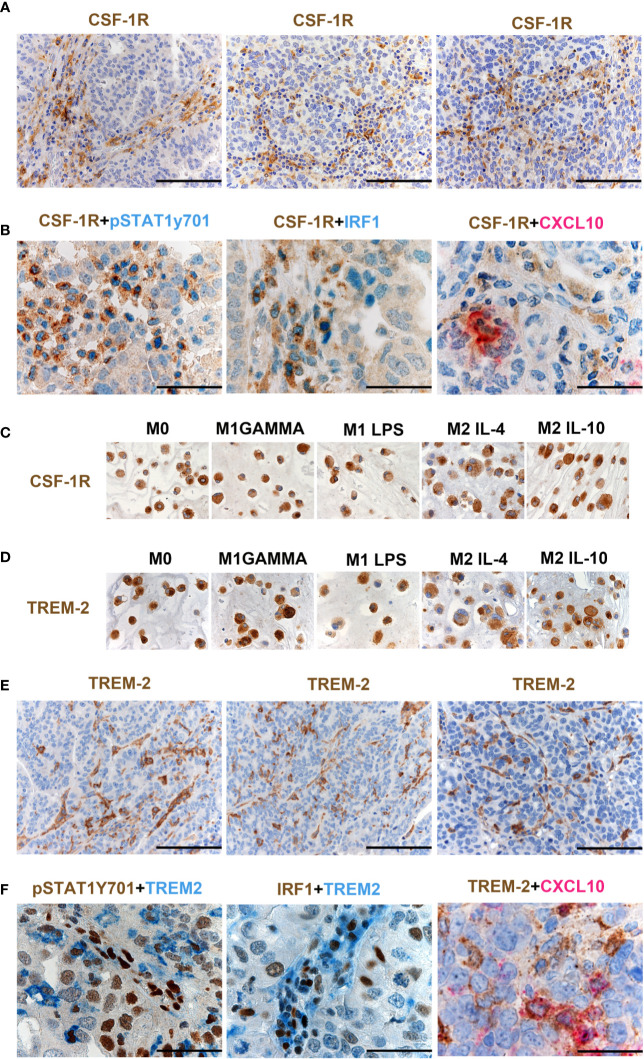
CSF1R and TREM-2 expression in OCs. Sections from HGSCs cases **(A, B, E, F)** and from cell-block preparations of polarized monocyte-derived Mϕ **(C, D)** immunostained as labeled. Magnification 200× (**A**, **E**; scale bar 100 um) and 400× (**B**, **F**, scale bar 50 um). CSF1R expression in OCs is largely restricted to TAMs **(A)**. Double immunohistochemistry showing co-expression of pSTAT1Y701, IRF1 and CXCL10 in CSF1R^+^ TAMs **(B)**. M1 type and M2 type Mϕ generated from peripheral blood monocytes express CSF1R **(C)**. M1 type Mϕ generated from peripheral blood monocytes express TREM2 **(D)**. TREM2 is expressed on TAMs in HGSCS. TREM2^+^ TAMs detected in ^Hi^OC-IS^30^CD163^Hi^ **(E)**; a fraction of TREM2^+^ TAMs co-expressed pSTAT1Y701, IRF1 and CXCL10 **(F)**.

As illustrated in **Supplementary Figure S4B**, CSF1 mRNA resulted significantly higher in HGSC compared to other OCs, and its level correlated with a high density of CD163^+^ TAMs (**Supplementary Table S6**), as also supported by *in vitro* findings documenting CD163 regulation by CSF1 (21). Moreover, CSF1R expression by Nanostring strongly correlates with CSF1R protein expression in OCs (**Figure 5B**). Previous studies have suggested expression of CSF1R on cancer cells in OCs (23), however, our findings clearly indicate that the expression is largely restricted to TAMs (**Figure 6A**). CSF1R blockade on TAMs has obtained some meaningful level of clinical efficacy in human cancer with high level of CSF1 (21). TAMs modulation by CSF1R blockade encompasses a range of biological activities from depletion to their reprogramming, the latter further amplified by CD40 agonist (24). ^Hi^OC-IS^30^CD163^Hi^ cases were significantly enriched in pSTAT1Y701^+^ TAMs (p = .007) as indicated in **Figures 5E, F**. By using double immunohistochemistry, we could detect a fraction CSF1R^+^ TAMs expressing pSTAT1Y701, IRF1 and CXCL10 (**Figure 6B**). Accordingly, also M1 type Mϕ generated from peripheral blood monocytes resulted CSF1R^+^ by IHC (**Figure 6C**). We have recently reported that TREM2 is selectively expressed on TAMs in various human cancer (25). TREM2 is expressed on CSF1R^+^ TAMs and is modulated by CSF1 and its blockade on TAMs results in delayed tumor growth, remodeling of the tumor immune contexture and increased ICI efficacy. We found that similarly to CD163 and CSF1R, TREM2 was also stably expressed by M1 type Mϕ generated from peripheral blood monocytes by IHC (**Figure 6D**). TREM2^+^ TAMs infiltrate ^Hi^OC-IS^30^CD163^Hi^ (**Figure 6E**), however, only a minor fraction of them co-expressed pSTAT1Y701, IRF1 and CXCL10 (**Figure 6F**). All these findings indicate that appropriate characterization of Mϕ on OCs requires modified approaches and might help in patient selection to CSF1R- and TREM2-blockade alone in combination with existing ICI.

### M1-Type Mϕ Polarization Occurs Across MϕSubsets and Cancer Types

We found that the prognostic power of ^Hi^OC-IS^30^ extend to various cancer types (**Figures 4D, E**). By using double immunohistochemistry for CD163 and pSTAT1Y701, we screened a set of human cancers including melanomas (n = 4), head and neck squamous cell carcinomas (n = 8), MSI^+^ colorectal carcinomas (n = 4), MSI^+^ endometrial carcinomas (n = 4), breast carcinomas (n = 8) and lung carcinomas (n = 4). A significant fraction of these cancers contained CD163^+^pSTAT1Y701^+^ M1-type Mϕ producing CXCL10 and surrounded by CD3 T-cell infiltration (**Supplementary Figures S8A, B**). These data extend our OCs findings across human cancer types.

**Figure 7:**
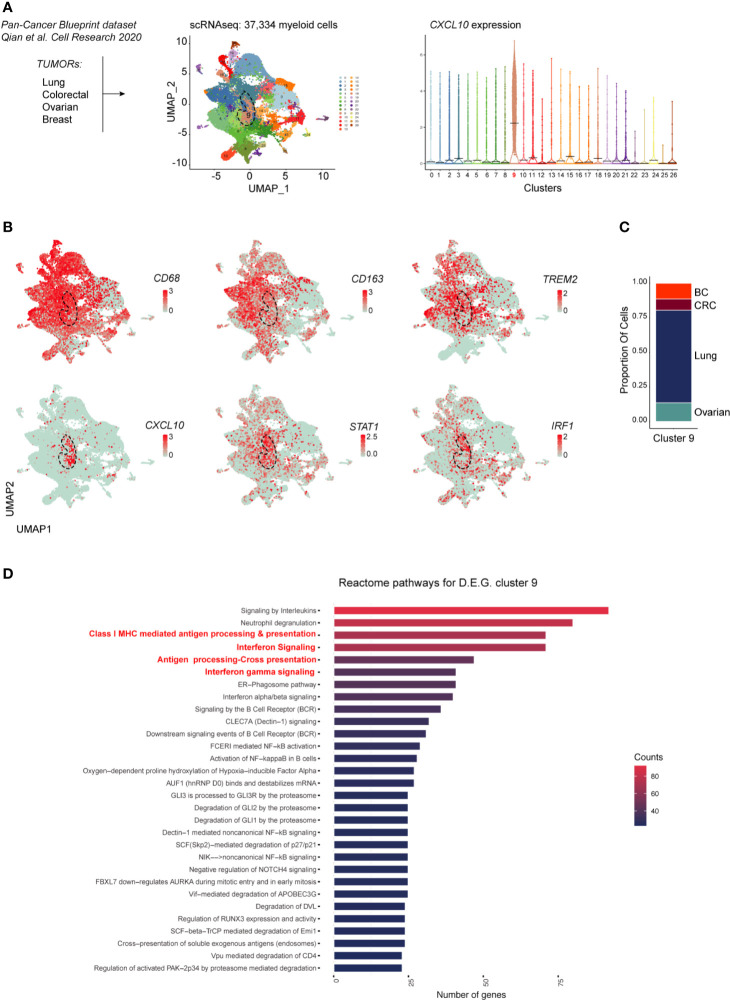
scRNAseq analysis of myeloid cells across cancer types. In **(A)** is shown the experimental design from published dataset: scRNAseq of myeloid cells (left panel). Dimensionality reduction of scRNAseq data merged from lung, colorectal, ovarian and breast tumors was performed using a Louvain graph-based clustering identifying 27 clusters (middle panel). Each dot represents an individual cell (n = 37,334). Violin plots illustrating expression distributions among the 27 clusters of CXCL10 (right panel). UMAP plot showing expression of CD68, CD163, TREM2, CXCL10, STAT1 and IRF1 **(B)**. Proportion of cells of the CXCL10+ cluster 9 per tumor type **(C)**; BC, breast cancer; CRC, colorectal cancer. Reactome pathway analysis for genes characterizing cluster 9 (adjusted P value <0.05) **(D)**.

Recent high dimensional studies of human tumor-associated myeloid cells have led to the identification of discrete TAM subsets based on their transcriptional profile. Specifically, emerging mononuclear phagocytes subsets in cancer are distinct on the basis of their ontogeny, differentiation state, functional orientation, proliferation potential and predictive power in response to ICI treatments (26, 27). To gain further insight on the transcriptional profile of M1-polarized TAMs in various cancer types we explored a pan-cancer scRNAseq dataset [n = 36; (19)] comprising ovarian HGSC, breast, lung and colorectal cancers. To this end, we merged 37,334 myeloid cells from all cancer types. Louvain Graph-based clustering at the resolution 0.6 identified 27 clusters of mononuclear phagocytes (**Figure 7A**). Among CD68^+^CD163^+^ TAMs also expressing the recently identified TREM2 marker, we could identify a CXCL10^+^IRF1^+^STAT1^+^ M1-type Mϕ population (Cluster 9) (**Figures 7A, B**) shared between all cancer types (**Figure 7C**). We next performed differential gene expression analysis between the CXCL10^+^IRF1^+^ TAM cluster (cluster 9) and the rest of the myeloid cell**s**. Gene pathway analysis showed that transcripts enriched in cluster 9 were involved in interferon signaling as well as in MHC-dependent antigen processing and in cross presentation (**Figure 7D** and **Supplementary Table S7**, **Supplementary Figure S9**). Altogether, these results show that M1-polarized TAMs form a functionally distinct subset of TAMs infiltrating various types of cancers. These M1-polarized TAMs are part of a T-cell infiltrated immune contexture positively correlating with better clinical outcome.

## Discussion

This study reports the characterization of the immune contexture in OCs, by digital microscopy analysis of a retrospective institutional cohort. Heterogeneity in terms of CD3^+^ T-cell and CD163^+^ Mϕ infiltration emerged among OC subtypes, including immune-infiltrated HGSC and T-cell poor CCC. Immune-infiltrated HGSC display high density of CD3^+^T-cells and of CD163^+^ TAMs associated with favorable clinical features and response to chemotherapy or platinum sensitivity. Gene expression analysis by using OC-IS^30^ immune signature generated from our institutional cohort and extended to the OV-TCGA dataset (6), uncovers the existence of a clinically meaningful functional immune response, particularly in the *BRCA* mutated subgroup. Immune-infiltrated HGSC contain CXCL10-producing IFNγ-polarized M1-type Mϕ surrounded by T-cells also expressing GZMB, indicating ongoing spontaneous T-cell response. All these findings were extended to and confirmed in other immunogenic human cancers types.

The clinical relevance of the endogenous immune response to ovarian carcinoma (OC), and specifically the favorable prognostic effect of CD3^+^ T-cells and CD8^+^ T-cells have been suggested by a set of observation from pre-clinical and clinical studies (13) and confirmed by a recent meta-analysis (15). Endogenous specific T-cell response has been documented in OCs. Neo-epitope specific CD8^+^ T-cells and CD4^+^ T-cells were identified both in peripheral blood and among TILs in immunotherapy-naïve OCs (28). Data on the role of Mϕ are less consistent. Early studies indicate that Mϕ purified from OCs ascites display functional heterogeneity (29), a finding consistent with distinct Mϕ polarizations associated to a bivalent behavior (30). In immune infiltrated OCs, the density of CD3^+^ T-cells correlates with the density of CD163^+^ TAMs and the two cell types resulted intermingled, suggesting their functional interaction. Of note, we found that a fraction of M1-type TAM in OCs produce abundant CXCL10, likely representing one of the relevant T-cell attracting chemokines in this neoplasm. To the other side of the spectrum, we identified a consistent subgroup of CCC containing macrophage deficient in M1-type polarization and lacking T-cell infiltration. CCC are distinct from HGSC in terms of molecular profile and response to systemic treatments; this study highlights distinct features also in terms of immune ecosystem likely accounting for their clinical behavior. Novel treatment options for CCC should consider these findings for a proficient bypass of the T-cell exclusion mechanisms.

The role of Mϕ in cancer immune surveillance is rapidly evolving (31, 32). In progressively growing cancer, TAMs modulate tumor progression by regulating various tumor-promoting functions including immunosuppression, angiogenesis, tumor cell proliferation, and stromal infiltration. However, recent findings indicate that similarly to other innate immune cells (33), human TAMs display a significant plasticity (34) as also confirmed by recent high dimensional analysis (26, 27). IFNγ-dependent M1 polarization can be mediated by neighboring T-cells, as observed in this study, or by NK cells (35). M1 Mϕ initiates pro-inflammatory responses and promotes direct or T-cell mediated antitumor effector functions (34, 36) particularly in highly immunogenic cancer (35). This plasticity accounts for a different prognostic relevance associated of TAMs. Based on this dichotomy, major approaches targeting these cells are exploring novel paradigms such as TAMs reprogramming in addition to their depletion and recruitment blockade, as for CSF1R blockade (21, 24). Various biomarkers have been proposed for the identification of TAMs polarization on archival tissue (37). By single-cell analysis of FFPE sections, this study identifies M1-type TAMs based on *in vitro* modeling of monocyte-derived M1^IFNγ^ and M1^IFNγ+LPS^. OCs-associated M1-type TAMs resulted CXCL10^+^IRF1^+^ STAT1p^+^. Data analysis of scRNAseq pan-cancer dataset confirmed the existence of a CXCL10^+^IRF1^+^ STAT1^+^ M1-type Mϕ across human cancers displaying activation of antigen presenting and cross presentation gene programs. IRF1 represents a crucial transcriptional regulator of the IFNγ-response (38) and recent findings on human cancers identified IRF1 as a central hub in cancer immunity (39). In macrophages, IRF1 drives M1 polarization (39) by increasing the expression of pro-inflammatory cytokines and chemokines (40). In addition, IRF1^+^ Mϕ displays a tumoricidal activity (41) mediated by nitric oxide. The microRNA (miRNA)-processing enzyme DICER is significantly down-modulated by IFNγ. Of note, STAT1^+^IRF1^+^ TAM have been observed in tumor-bearing mice with DICER conditional deletion (42) and resulted in tumor inhibition by recruitment of activated CTL.

Immune infiltrated HGSC are defined by CD3^Hi^CD163^Hi^ immunoscore and display a better outcome, independently from other major prognosticators. Immune infiltrated HGSC are also enriched of OC-IS^30^. The immune cell component plays a relevant role in the clinical response to various HGSC treatments (43). The primary systemic treatments include chemotherapy with platinum-based regimens combined with taxanes. Of note, outcomes of platinum-based regimen are significantly dependent on the existing tumor immune microenvironment (44). In the last few years, Poly (ADP-ribose) polymerase (PARP) inhibitors have been included for HGSC showing HRD. Several trials demonstrated the efficacy of these compounds both as maintenance therapy after first-line chemotherapy (8, 45) or after the treatment of recurrent disease. The best performance for PARP inhibitors is observed in tumors with *BRCA1* or *BRCA2* mutation or with at least an HRD phenotype. A recent meta-analysis confirmed their efficacy with improvement of PFS in platinum-sensitive recurrent OC (7). The findings presented here indicate that a proficient immune microenvironment predicts a better outcome. *BRCA1* and *BRCA2* mutated tumors are also densely infiltrated by T-cells, however, we found that the prognostic effect of the OC-IS^30^ signature, as tested in the TCGA cohort, is independent and additive from *BRCA* status and others prognosticator (**Figure 4B**).

The role of immunotherapy in OCs has been recently investigated by testing the efficacy of ICI (anti-PD1 or anti-PDL1) as single therapy. The results of the first trials with ICIs (46) showed a fair effectiveness. However, the recent combination of ICIs and PARPi provided better results (11, 47). The best results obtained applying ICIs in the subgroup of *BRCA1* or *BRCA2* mutated patients can be explained by recent studies showing that PD1 and PDL1 are highly expressed *in BRCA1* or *BRCA2* mutated patients. Moreover, PARPi administration to breast cancer cell lines further enhance PD-L1 by inactivating GSK3β (48), thus explaining the benefit obtained by the combination of PARPi and anti-PD-L1 therapy (11). It should be reminded that, particularly in HGSC, PD-L1 is primarily expressed by macrophages and that a high density of PD-L1^+^ Mϕ correlates with CD8^+^ T-cells and predicts favorable survival (49). The cellular source and the magnitude of expression of PD-L1 might variably dictate its immune escape potency (50). Based on our findings, it is highly likely that the major source of PD-L1 in OC is from innate immune resistance mechanisms with its dominant hub on M1-type TAMs, whose fine-tuned modulation might further enhance the clinical benefit. These findings identify the combined analysis of immune-contexture and immune signatures as a novel biomarker in OCs management, to be further investigated in the predictive setting.

In conclusion, the results of this study document a proficient immune contexture in a subgroup of primary OCs. Findings proposed here are in keeping with a relevant role of the innate TAMs compartment in OCs immune surveillance, likely unleashing the endogenous adaptive T-cell response. However, T-cell exclusion occurs also in OCs, particularly in the CCC subtype, likely as a result of the lack of CXCL10^+^-producing M1-type Mϕ. Since CCC is already infiltrated by macrophages, their repolarization to a CXCL10^+^TAM might provide a clinical benefit. As an extension of this analysis, M1-type Mϕ sharing a common transcriptional activation state were also detected across various human immunogenic cancers. However, intratumor heterogeneity in TAM polarization emerged in this study, with also a fraction of CSF1R and TREM2 M1-type Mϕ. This indicates that using approaches targeting molecules of immunosuppressive myeloid cells such as CSF1R and TREM2 would partially affect the endogenous anti-tumor TAM component. Instead, implementation of reprogramming approaches that further bolster the already present macrophage component is needed.

## Data Availability Statement

The original contributions presented in the study are included in the article/**Supplementary Material**. Further inquiries can be directed to the corresponding author.

## Ethics Statement

The study was approved by the local IRB, WW-IMMUNOCANCERhum, NP-906 and NP-1284. Written informed consent for participation was not required for this study in accordance with the national legislation and the institutional requirements.

## Author Contributions

LA, FM, MB, LB, and WV contributed to conception and design of the study. LZ, EB, AR, GT, and FO organized the clinical database. MB, LB, IP, MM, SG, and YM-K performed experiments. FM and SC performed the statistical analysis. LA, FM, MB, LB, and WV wrote the first draft of the manuscript. CR and JH wrote sections of the manuscript. All authors contributed to the article and approved the submitted version.

## Conflict of Interest

SG was employed by Diatech Pharmacogenetics srl and CR was employed by Dr Carola Ries Consulting.

The remaining authors declare that the research was conducted in the absence of any commercial or financial relationships that could be construed as a potential conflict of interest.
