# Supplementary material for: Infiltration by CXCL10 Secreting Macrophages Is Associated With Antitumor Immunity and Response to Therapy in Ovarian Cancer Subtypes
Source: Front Immunol. 2021 Jun 18;12:690201. doi: 10.3389/fimmu.2021.690201 (PMC8253056; doi:10.3389/fimmu.2021.690201)
Supplement: Supplementary file 1 [file DataSheet_1.pdf]

Infiltration by CXCL10 secreting macrophages is associated with antitumor immunity and response to therapy in ovarian cancer subtypes

Laura Ardighieri†[1], Francesco Missale†[2,3], Mattia Bugatti†[1,2], Luisa Benerini Gatta[2], Irene Pezzali[2], Matilde Monti[2], Stefano Gottardi[4], Laura Zanotti[5,6], Eliana Bignotti[5,6], Antonella Ravaggi[5,6,7], Germana Tognon[6], Franco Odicino[6,7], Stefano Calza[8,9,10], Yoann Missolo-Koussou[11], Carola Hermine Ries[12], Julie Helft[11] and William Vermi*[1,2,13]

[1] Unit of Pathology, ASST Spedali Civili di Brescia, 25100 Brescia, Italy

[2] Department of Molecular and Translational Medicine, University of Brescia, 25125 Brescia, Italy

[3] IRCCS Ospedale Policlinico San Martino, 16121 Genova, Italy

[4] Diatech Pharmacogenetics srl, 60035 Jesi, Italy

[5] "Angelo Nocivelli" Institute of Molecular Medicine, ASST Spedali Civili of Brescia- University of Brescia, 25123 Brescia, Italy

[6] Division of Obstetrics and Gynecology, ASST Spedali Civili di Brescia, Brescia, Italy

[7] Department of Clinical and Experimental Science, University of Brescia, 25125 Brescia, Italy

[8] Unit of Biostatistics, Department of Molecular and Translational Medicine, University of Brescia, 25125 Brescia, Italy

[9] Department of Medical Epidemiology and Biostatistics, Karolinska Institutet, 17177 Stockholm, Sweden

[10] Big & Open Data Innovation Laboratory, University of Brescia, 25125 Brescia, Italy

[11] PSL University, Institut Curie Research Center, INSERM U932 & SiRIC, Center for cancers immunotherapy, Translational Immunotherapy Team, F-75005, Paris, France.

[12] Dr. Carola Ries Consulting, 82377 Penzberg, Germany

[13] Department of Pathology and Immunology, Washington University School of Medicine, St. Louis, MO 63130, USA

* Correspondence: william.vermi@unibs.it; phone:+39-030-399-8425

† These authors equally contributed to the work.

Running title: Immune-contexture in ovarian cancer

Keywords: T-cells, macrophages, ovarian cancer, signature, interferon gamma

Supplementary Figures

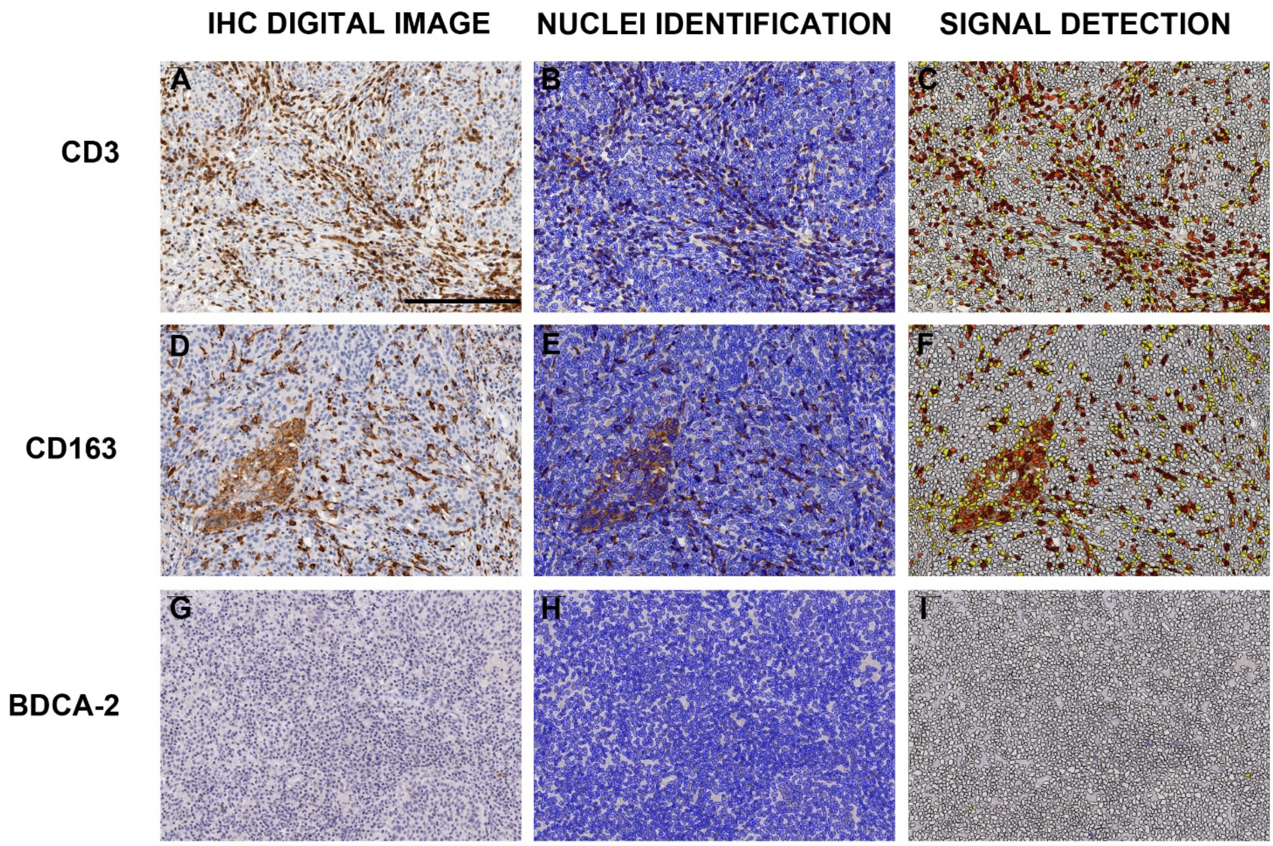

Supplementary Figure S1. Digital image analysis of T-cells, tumor-associated macrophages (TAMs) and Plasmacytoid dendritic cells (PDCs) in ovarian carcinoma. Sections are from one representative HGSC case. **A, D, G**: digitalized immunostained slides (200x magnification; scale bar 200 um) recognizing $CD3^+$ T-cells (**A**), $CD163^+$ TAMs (**D**) and $BDCA2^+$ PDCs (**G**). **B, C, E, F, H, I**: absolute immune cells count performed with Tissue Studio 2.0, based on segmentation of nucleus objects (**B, E, H**) and recognition of positive intraepithelial and stromal immune cells (**C, F, I**), with different staining intensity (yellow mild, red moderate and brown strong).

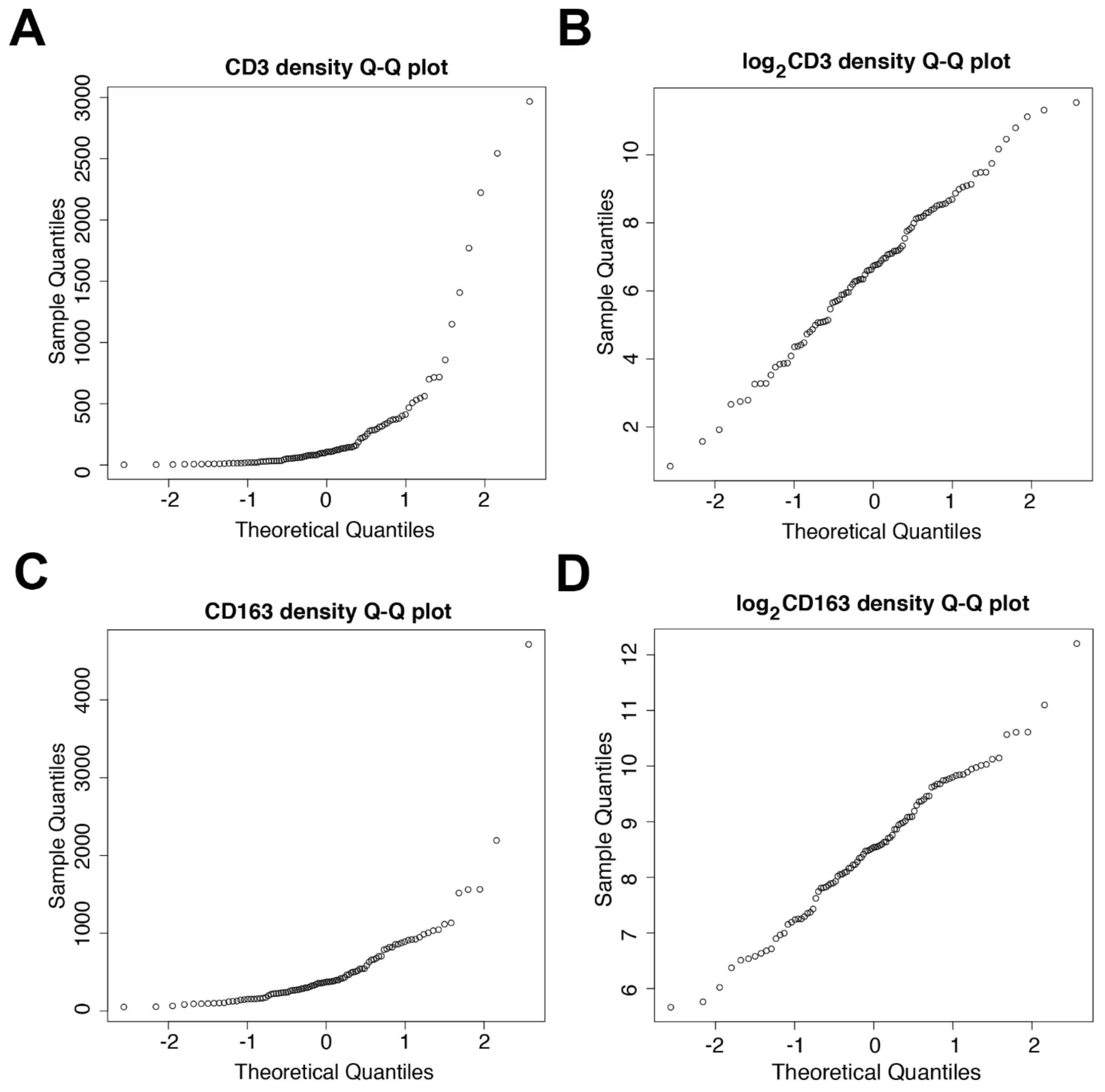

Supplementary Figure S2. **Distribution of immune cell variables.** Q-Q plots of $CD3^+$, log_2CD3^+, $CD163^+$ and log_2CD163^+ cells densities showing that both immune populations densities follow log-normal distributions.

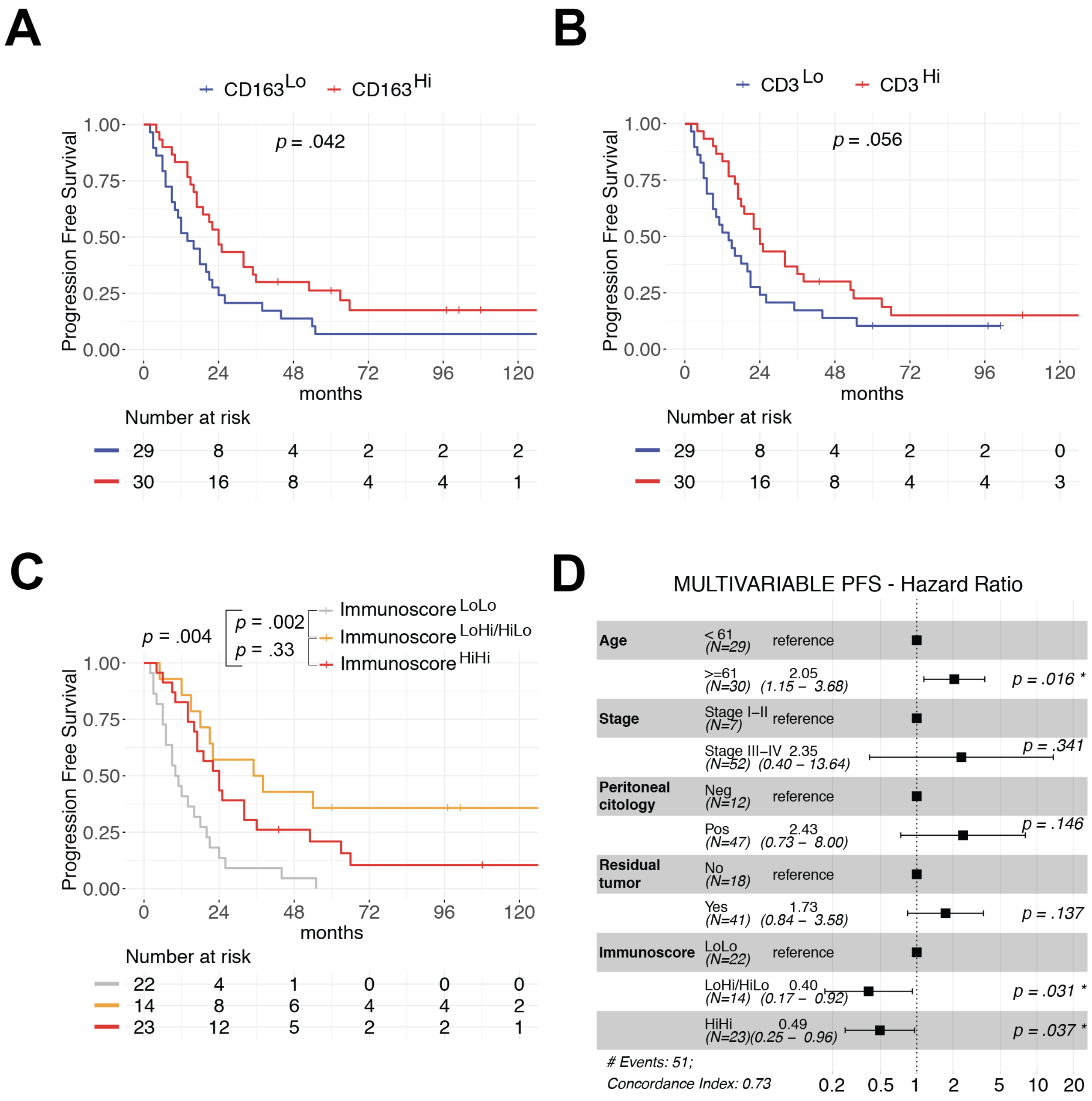

Supplementary Figure S3. **Progression free survival and immune contexture.** Univariable analysis of progression free survival estimates (Kaplan-Meier method) according to high density of CD3^{+} T-cells (**A**), high density of CD163^{+} TAMs (**B**) or Immunoscore classes (**C**). P values estimated with log-rank test (**A, B**), pairwise comparisons p-values adjusted with FDR (**C**). Forest-plot reporting H.R. with $CI_{95\%}$ of multivariable progression free survival analysis with Cox-model (**D**).

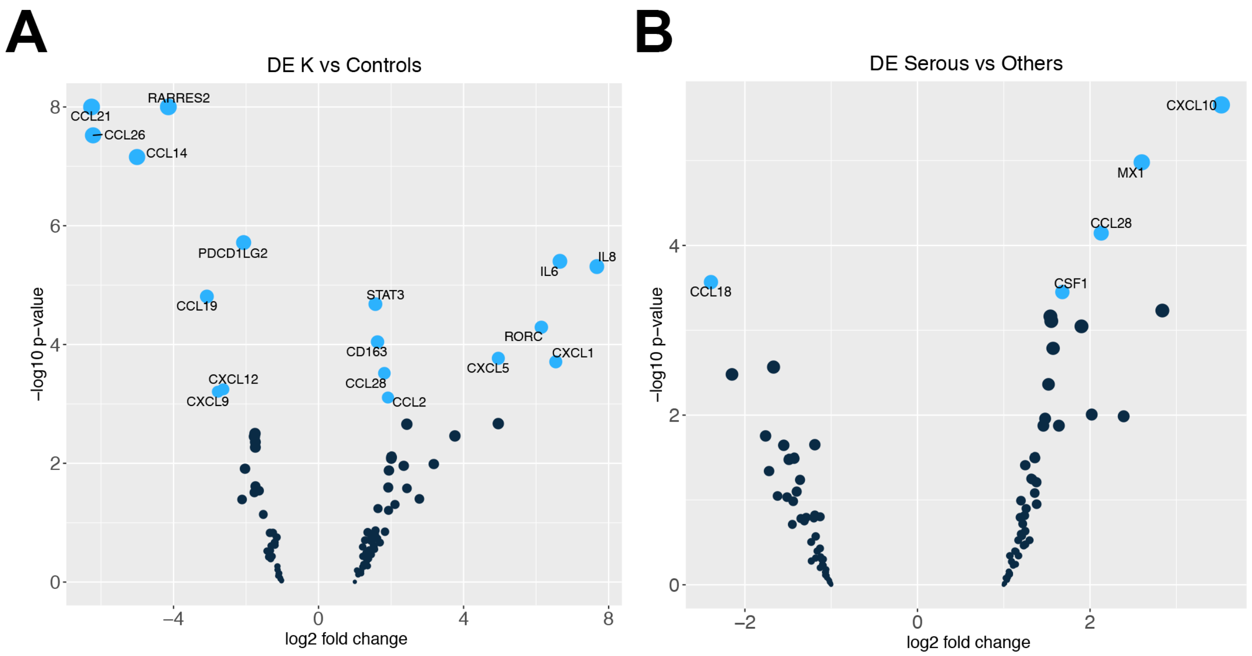

Supplementary Figure S4. Differential gene expression analysis. Volcano plots showing differentially expressed genes in patients affected by ovarian cancer, compared to healthy ovarian controls (**A**), and differentially expressed genes in HGSC compared to EC or CCC (**B**). Light blue dots representing significant different expressed genes by FDR adjustment.

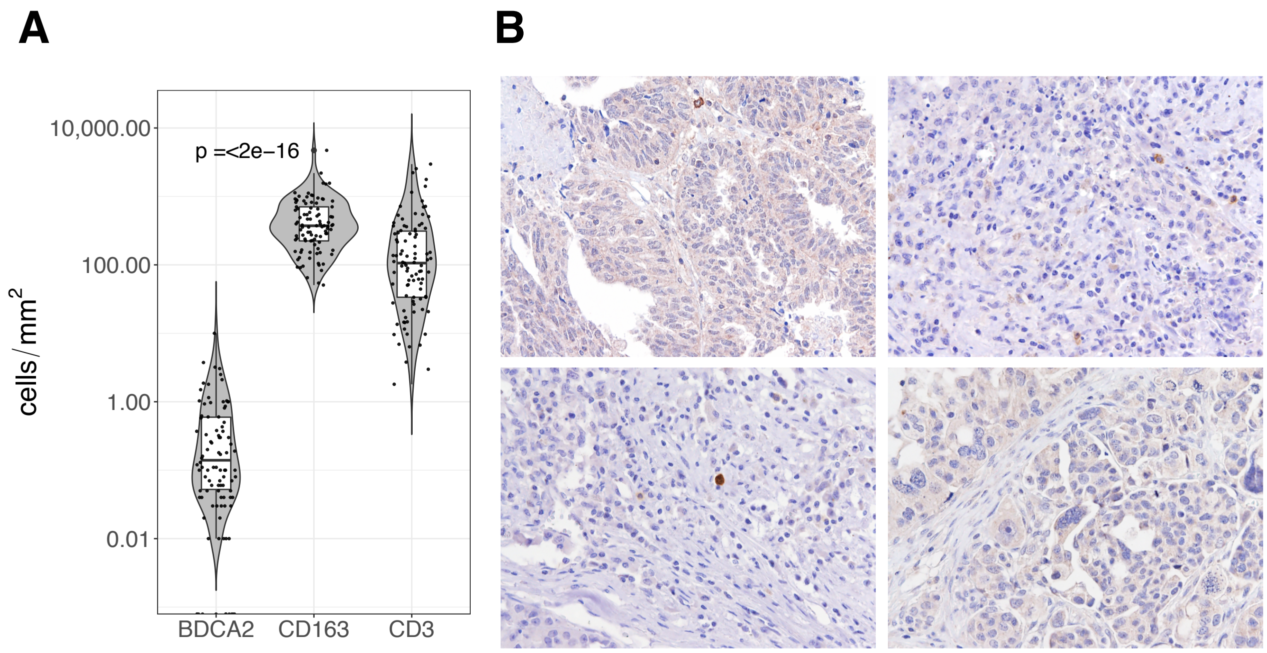

Supplementary Figure S5. Limited infiltration of plasmacytoid dendritic cells in OCs. Violin plots of immune cells distribution in OCs sections, p value estimated by ANOVA (**A**); representative sections from OCs cases immunostained for BDCA2 showing the rare presence of BDCA2^{+} PDCs (**B**). Magnification 200x.

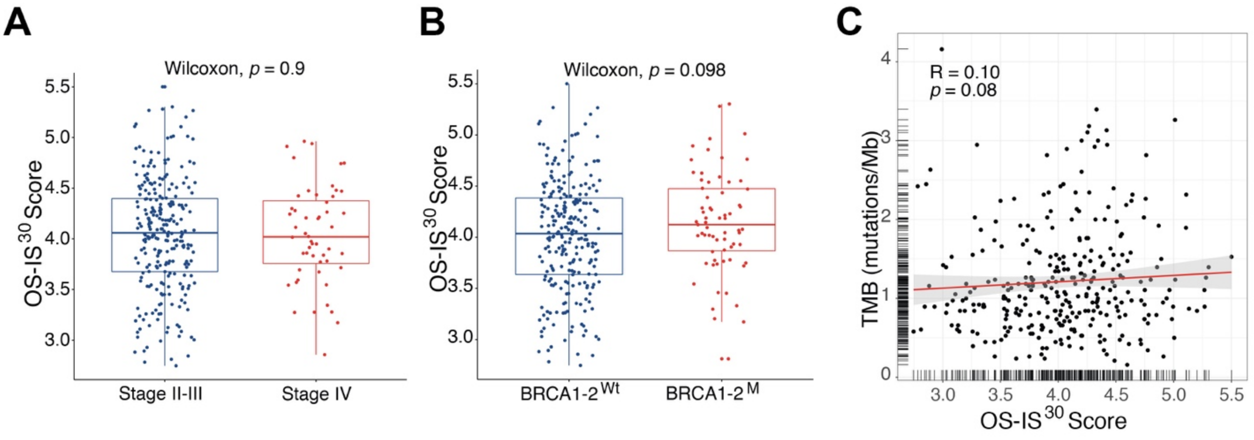

Supplementary Figure S6. Clinical correlates of OC-IS^{30}. Box plots showing OC-IS^{30} score in different tumor stages groups (**A**), BRCA1 and BRCA2 mutational status groups (**B**) and scatter plot showing no correlation between OC-IS^{30} score and TMB (**C**). P values estimated by Wilcoxon unpaired test in A, B and Spearman test in C.

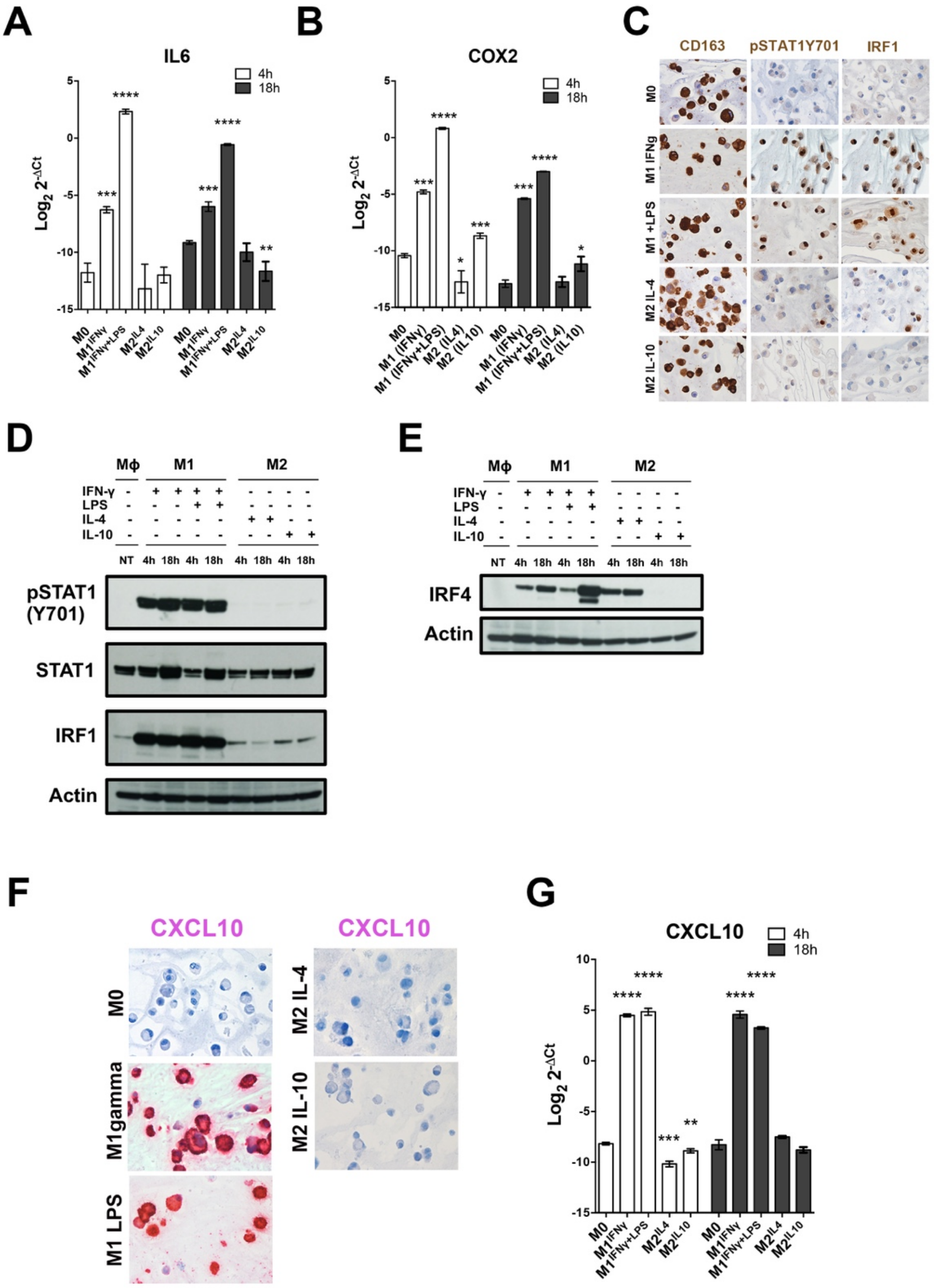

Supplementary Figure S7. Macrophage polarization in OCs. In **A, B** and **G** the IL6, COX2 and CXCL10 genes expression was measured by qRT-PCR on polarized monocyte-derived Mϕ, IFNa (100 U/mL) unstimulated or stimulated with IFN-γ (50 ng/mL) or IFN-γ (20 ng/mL) + LPS (100 ng/mL) or IL-4 (20 ng/mL) or IL-10 (20 ng/mL) for 4 or 18 hours. The mRNA relative expression (-ΔCt) was normalized to the housekeeping gene using the comparative Ct method. The mean and SD of technical replicates (n = 3) are shown. The statistical significance was calculated by a Student's t test. * $p < 0.05$; ** $p < 0.01$; *** $p < 0.001$; **** $p < 0.0001$. In **C** sections from cell-block preparations of polarized monocyte-derived Mϕ subjected to *in situ* hybridization as labeled. Magnification 400X. Western blot analysis performed on polarized monocyte-derived Mϕ illustrate the protein expression of IRF1, pSTAT1Y701 and IRF4 as labeled (**D, E**). Sections from cell-block preparations of polarized monocyte-derived Mϕ subjected to *in situ* hybridization as labeled. Magnification 400X (**F**).

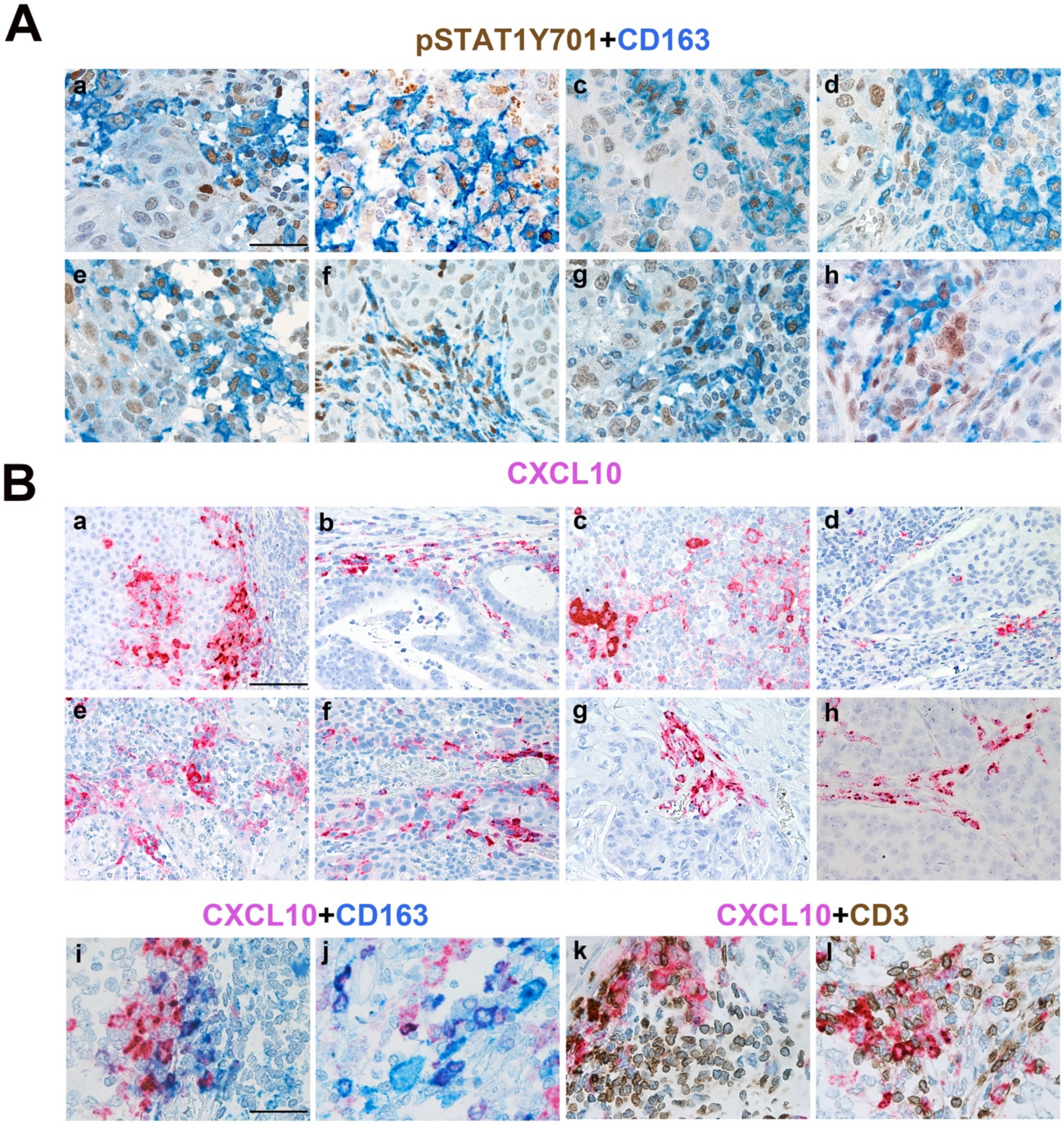

Supplementary Figure S8. M1-type Mϕ polarization across cancer types. Sections from various cancer types (**A, B**) immunostained as labeled. Magnification 200X (B: a-h; scale bar 100 um) and 400X (A, B: i-l, scale bar 50 um). Sections from various cancer types showing the presence of $CD163^{+}pSTAT1Y701^{+}$ (**A**, **a** melanomas, **b** colorectal carcinoma, **c** endometrial carcinoma, **d** lung carcinoma, **e** oral squamous cell carcinomas, **f** laryngeal carcinoma, **g** triple negative breast carcinoma, **h** $HER2^{+}$ breast carcinoma). CXCL10 is abundantly produced in different tumor types (**B**, **a** melanomas, **b** colorectal carcinoma, **c** endometrial carcinoma, **d** lung carcinoma, **e** oral squamous cell carcinomas, **f** laryngeal carcinoma, **g** triple negative breast carcinoma, **h** $HER2^{+}$ breast carcinoma). and it is expression is detected in $CD163^{+}$ TAMs (**B: i** and **k** melanoma; **j** colorectal carcinoma, **l** endometrial carcinoma).

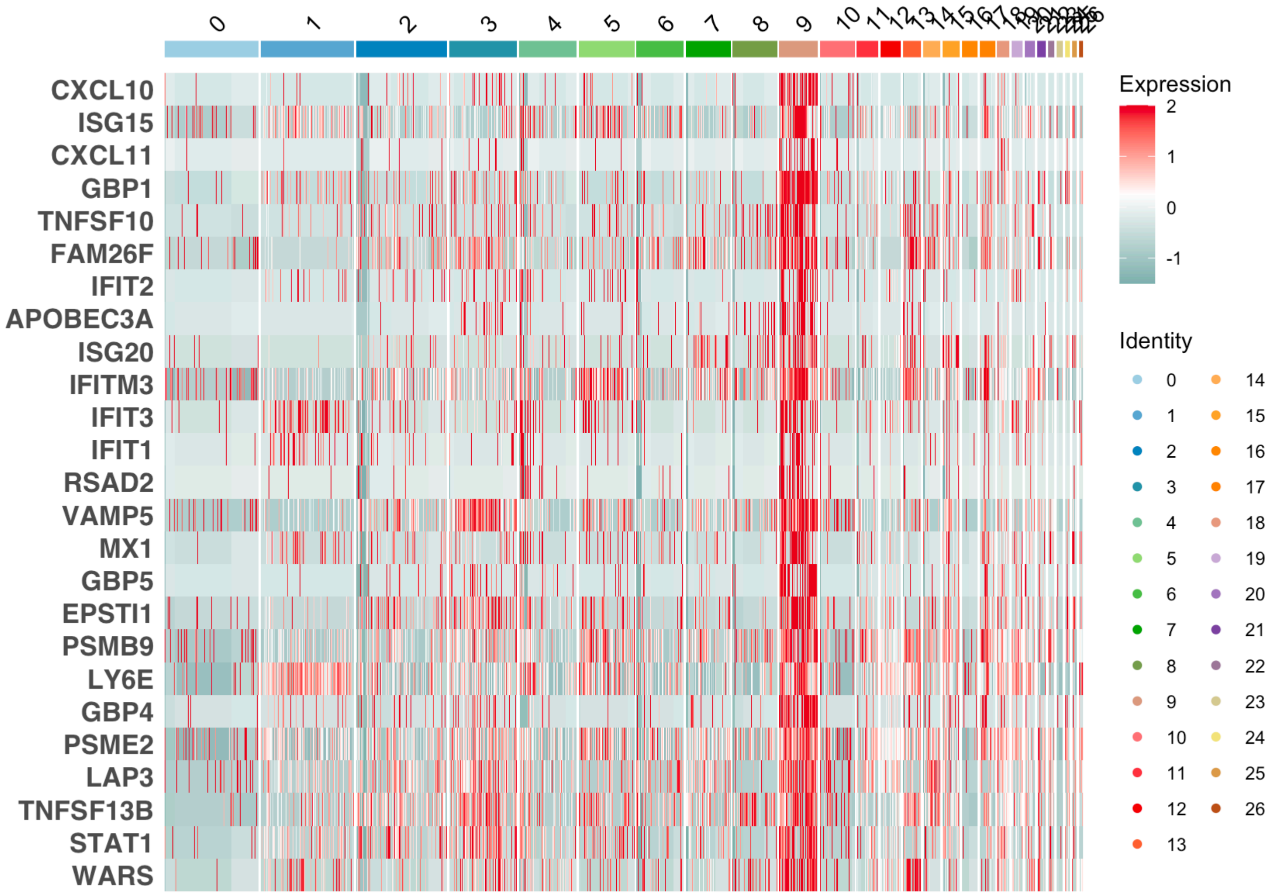

Supplementary Figure S9: scRNAseq analysis of myeloid cells across cancer types. Heatmap showing normalized expression of the top 25 most variable genes in cluster 9, as defined in Figure 9A.
