# Supplementary material for: Infiltration by CXCL10 Secreting Macrophages Is Associated With Antitumor Immunity and Response to Therapy in Ovarian Cancer Subtypes
Source: Front Immunol. 2021 Jun 18;12:690201. doi: 10.3389/fimmu.2021.690201 (PMC8253056; doi:10.3389/fimmu.2021.690201)
Supplement: Supplementary file 2 [file DataSheet_2.pdf]

Infiltration by CXCL10 secreting macrophages is associated with antitumor immunity and response to therapy in ovarian cancer subtypes

Laura Ardighieri†[1], Francesco Missale†[2,3], Mattia Bugatti†[1,2], Luisa Benerini Gatta[2], Irene Pezzali[2], Matilde Monti[2], Stefano Gottardi[4], Laura Zanotti[5,6], Eliana Bignotti[5,6], Antonella Ravaggi[5,6,7], Germana Tognon[6], Franco Odicino[6,7], Stefano Calza[8,9,10], Yoann Missolo-Koussou[11], Carola Hermine Ries[12], Julie Helft[11] and William Vermi*[1,2,13]

[1] Unit of Pathology, ASST Spedali Civili di Brescia, 25100 Brescia, Italy

[2] Department of Molecular and Translational Medicine, University of Brescia, 25125 Brescia, Italy

[3] IRCCS Ospedale Policlinico San Martino, 16121 Genova, Italy

[4] Diatech Pharmacogenetics srl, 60035 Jesi, Italy

[5] "Angelo Nocivelli" Institute of Molecular Medicine, ASST Spedali Civili of Brescia-University of Brescia, 25123 Brescia, Italy

[6] Division of Obstetrics and Gynecology, ASST Spedali Civili di Brescia, Brescia, Italy

[7] Department of Clinical and Experimental Science, University of Brescia, 25125 Brescia, Italy

[8] Unit of Biostatistics, Department of Molecular and Translational Medicine, University of Brescia, 25125 Brescia, Italy

[9] Department of Medical Epidemiology and Biostatistics, Karolinska Institutet, 17177 Stockholm, Sweden

[10] Big & Open Data Innovation Laboratory, University of Brescia, 25125 Brescia, Italy

[11] PSL University, Institut Curie Research Center, INSERM U932 & SiRIC, Center for cancers immunotherapy, Translational Immunotherapy Team, F-75005, Paris, France.

[12] Dr. Carola Ries Consulting, 82377 Penzberg, Germany

[13] Department of Pathology and Immunology, Washington University School of Medicine, St. Louis, MO 63130, USA

* Correspondence: william.vermi@unibs.it; phone:+39-030-399-8425

† These authors equally contributed to the work.

Running title: Immune-contexture in ovarian cancer

Keywords: T-cells, macrophages, ovarian cancer, signature, interferon gamma

Supplementary Tables

Supplementary Table S1: Full database of the OC cohort considered (attached excel file))

Supplementary Table S2: Nanostring probes details (attached excel file)

Supplementary Table S3. Gene Expression Assays.

Gene	Cat. no.	Company
**COX2/PTGS2**	Hs00153133_m1	Thermo Fisher Scientific
**CXCL10**	Hs00171042_m1	Thermo Fisher Scientific
**IL-6**	Hs00174131_m1	Thermo Fisher Scientific
**RPS18**	qHsaCEP0040177	Bio-Rad

Supplementary Table S4: Western blotting antibodies.

Antibody	Clone	Isotype	Company	Dilution
Actin	polyclonal	Rabbit	Sigma – Aldrich	1:1000
IRF-1	D5E4	Rabbit	Cell Signaling	1:1000
IRF-4	E8H3S	Rabbit	Cell Signaling	1:1000
pStat1 (Tyr701)	58D6	Rabbit	Cell Signaling	1:1000
Stat1	polyclonal	Rabbit	Cell Signaling	1:1000

Supplementary Table S5: Univariable survival analysis of HGSC

Variables		N. (%)	Overall survival		Progression free survival	
			H.R. (95% CI)	p	H.R. (95% CI)	p
Age	< median	29 (49%)	1	0.204	1	0.133
	>= median	30 (51%)	1.48 (0.81 - 2.73)		1.53 (0.88 - 2.65)	
Menopause	no	16 (27%)	1	0.93	1	0.937
	yes	43 (73%)	0.97 (0.5 - 1.87)		0.98 (0.53 - 1.79)	
FIGO Stage	I-II	7 (12%)	1	**0.0203**	1	**0.009**
	III-IV	52 (88%)	10.56 (1.44 - 77.3)		6.63 (1.6 - 27.45)	
Residual Tumor	no	18 (31%)	1	**< 0.001**	1	**0.002**
	yes	41 (69%)	7.15 (2.75 - 18.64)		2.89 (1.47 - 5.68)	
Peritoneal cytology	negative	12 (20%)	1	**0.007**	1	**0.002**
	positive	47 (80%)	4.25 (1.5 - 12.08)		4.41 (1.72 - 11.32)	
CD3	Low	29 (49%)	1	**0.001**	1	0.056
	High	30 (51%)	0.35 (0.19 - 0.66)		0.58 (0.34 - 1.01)	
CD163	Low	29 (49%)	1	**0.019**	1	**0.042**
	High	30 (51%)	0.48 (0.26 - 0.89)		0.56 (0.32 - 0.98)	
Immunoscore	LoLo	22 (37%)	1		1	
	LoHi/HiLo	14 (24%)	0.17 (0.07 - 0.43)	**< 0.001**	0.23 (0.1 - 0.51)	**< 0.001**
	HiHi	23 (39%)	0.28 (0.14 - 0.56)	**< 0.001**	0.41 (0.22 - 0.76)	**0.005**

Supplementary Table S6: OC-IS[30] Gene signature, R and adjusted p-values (Hommel) of Pearson correlation analysis between log_2gene expression and log_2Immune cell densities. β coefficients obtained by ridge linear regression of Z-score gene expression having as dependent variable the sum of $CD3^+$ and $CD163^+$ cell densities.

Gene	CD3		CD163		CD3 + CD163
	R	p	R	p	β
CXCL10	0.73	2.3E-12	0.58	2.9E-06	0.0574
IRF1	0.71	2.4E-11	0.58	3.1E-06	0.0573
CSF1	0.48	0.0009	0.51	0.0002	0.0573
CXCL9	0.76	4.6E-14	0.54	3.6E-05	0.0506
IL15	0.50	0.0003	0.43	0.0087	0.0385
PDCD1LG2	0.71	3.4E-11	0.54	4.8E-05	0.0357
STAT1	0.54	4.1E-05	0.50	0.0004	0.0333
GZMA	0.75	1.1E-13	0.52	0.0001	0.0320
CD274	0.76	4.2E-14	0.53	5.4E-05	0.0310
CCL4	0.41	0.0215	0.38	0.0701	0.0284
CCL2	0.39	0.0431	0.28	0.9895	0.0282
OAS1	0.44	0.0079	0.43	0.0105	0.0267
CD8A	0.61	2.7E-07	0.46	0.0027	0.0254
MX1	0.39	0.0497	0.47	0.0017	0.0243
CXCL16	0.39	0.0414	0.44	0.0059	0.0209
CXCL13	0.58	2.0E-06	0.46	0.0027	0.0206
GNLY	0.64	3.7E-08	0.49	0.0007	0.0197
CCL8	0.44	0.0065	0.36	0.1310	0.0191
IDO1	0.58	2.7E-06	0.43	0.0114	0.0167
CSF1R	0.45	0.0040	0.42	0.0169	0.0167
GZMH	0.57	5.7E-06	0.38	0.0618	0.0143
CD86	0.41	0.0205	0.32	0.4621	0.0139
CCL5	0.75	2.1E-13	0.60	7.7E-07	0.0116
CD163	0.41	0.0193	0.41	0.0232	0.0102
GZMB	0.60	9.2E-07	0.40	0.0344	0.0095
PRF1	0.61	2.8E-07	0.45	0.0051	0.0093
CTLA4	0.57	7.4E-06	0.35	0.1727	0.0092
CXCL11	0.65	8.9E-09	0.44	0.0069	0.0086
CCL19	0.50	0.0004	0.30	0.7086	0.0013
PDCD1	0.41	0.0187	0.31	0.6830	0.0006

Supplementary Table S7: Results of the gene pathway analysis investigating the cluster 9, identified by scRNAseq analysis, through the Reactome datasets (attached .csv file).
